# Triple negative breast cancer metastasis is hindered by a peptide antagonist of F11R/JAM‑A protein

**DOI:** 10.1186/s12935-023-03023-4

**Published:** 2023-08-11

**Authors:** Radosław Bednarek, Dagmara W. Wojkowska, Marcin Braun, Cezary Watala, Moro O. Salifu, Maria Swiatkowska, Anna Babinska

**Affiliations:** 1 Department of Cytobiology and Proteomics, Chair of Biomedical Sciences, Medical University of Lodz, ul. Mazowiecka 6/8, 92-215 Lodz, Poland; 2 Department of Haemostasis and Haemostatic Disorders, Medical University of Lodz, Lodz, Poland; 3 Department of Pathology, Chair of Oncology, Medical University of Lodz, Lodz, Poland; 4 Department of Medicine, Downstate Medical Center, State University of New York, Brooklyn, NY USA

**Keywords:** Triple-negative breast cancer, Metastasis, Endothelial barrier, Epithelial barrier, Tight junctions, Mouse breast cancer model, F11R/JAM-A, F11R/JAM-A-derived peptide

## Abstract

**Background:**

The F11R/JAM-A cell adhesion protein was examined as the therapeutic target in triple negative breast cancer (TNBC) with the use of the peptide antagonist to F11R/JAM-A, that previously inhibited the early stages of breast cancer metastasis in vitro.

**Methods:**

The online in silico analysis was performed by TNMPlot, UALCAN, and KM plotter. The in vitro experiments were performed to verify the effect of peptide 4D (P4D) on human endothelial cell lines EA.hy926 and HMEC-1 as well as on human TNBC cell line MDA-MB-231. The cell morphology upon P4D treatment was verified by light microscopy, while the cell functions were assessed by colony forming assay, MTT cell viability assay, BrdU cell proliferation assay, and Transepithelial/Endothelial Electrical Resistance measurements. The in vivo experiments on 4T1 murine breast cancer model were followed by histopathological analysis and a series of quantitative analyses of murine tissues.

**Results:**

By in silico analysis we have found the elevated gene expression in breast cancer with particular emphasis on TNBC. The elevated F11R expression in TNBC was related with poorer survival prognosis. Peptide 4D has altered the morphology and increased the permeability of endothelial monolayers. The colony formation, viability, and proliferation of MDA-MB-231 cells were decreased. P4D inhibited the metastasis in 4T1 breast cancer murine model in a statistically significant manner that was demonstrated by the resampling bootstrap technique.

**Conclusions:**

The P4D peptide antagonist to F11R/JAM-A is able to hinder the metastasis in TNBC. This assumption needs to be confirmed by additional 4T1 mouse model study performed on larger group size, before making the decision on human clinical trials.

**Supplementary Information:**

The online version contains supplementary material available at 10.1186/s12935-023-03023-4.

## Background

Breast cancer (BC) is the most frequently diagnosed cancer in women and the leading cause of cancer death among females [1, 2]. BC is a heterogeneous disease with 5 subtypes identified due to Ki67 index, estrogen receptor (ER), progesterone receptor (PR) and human epidermal growth factor receptor 2 (HER2/erbB2) expression: 2 ER-positive luminal subtypes (luminal A: low Ki67 level, and luminal B: high Ki67 level) and 3 ER-negative subtypes (HER2-enriched, basal-like/triple-negative and normal-like) [1, 3]. Triple-negative breast cancer (TNBC) remains among the worst prognostic and the most aggressive subtypes of BC [4]. Due to its highest aggressiveness and heterogeneity of all BC subtypes TNBC has been described as fatal, being reportedly the greatest cause of mortality in women [5]. TNBC accounts for about 10–15% of all diagnosed BC cases [6]. Although TNBC is the subtype with the most complete response to chemotherapy (22%), the recurrence and metastasis rate of TNBC patients is higher than other BC subtypes [7]. The heterogeneity and invasiveness of TNBC contribute to relapse or metastasis in the early stage of its development [4]. TNBC is usually associated with metastasis to the following organs: the brain, lungs, bones, and liver, with an average survival time of 18 months [5]. Due to the lack of ER, PR and HER2/erbB2 expression the treatment options for TNBC are limited to conventional chemotherapies rather than molecular targeted therapies [7]. Recently, several new targeted therapies have emerged and have been approved, including poly (ADP-ribose) polymerase (PARP) inhibitors, for example olaparib (approved in 2018), and talazoparib (approved in 2019), recommended for patients with BRCA mutations [8]. Other therapies for TNBC approved by FDA include the programmed cell death ligand 1 (PD-L1) inhibitor atezolizumab [9], the anti-trophoblast cell-surface antigen-2 (Trop-2) antibody drug conjugate (ADC) sacituzumab govitecan [10], as well as the phosphoinositide-3-kinase–protein kinase B/Akt (PI3K-PKB/Akt) pathway inhibitors, including alpelisib, ipatasertib, and capivasertib [11]. Another potentially promising strategy in TNBC treatment seems to be the targeting dormant cancer stem cells; however, it is impossible to evaluate the efficacy of this dormant cell–killing approach in patients, since currently there is no available diagnostic tools for dormant cell detection [12]. There are some other limitations for most of the targeted therapies for TNBC: the durable responses are usually not achievable and some patients may experience serious adverse events, including neutropenia and peripheral neuropathy [8]. These poor outcomes and therapy limitations highlight an ongoing need to identify new targets and/or new compounds with potential therapeutic properties in TNBC [13].

The aberrant expression of a tight junction (TJ) protein known as F11 platelet receptor aka junctional adhesion molecule-A (F11R/JAM-A) is linked with most cancer types, particularly with breast cancer [14–17]. The F11R/JAM-A overexpression in breast tumor tissue was associated with aggressive cancer phenotypes [15, 17, 18]. F11R/JAM-A is a direct target of transcriptional regulation by miR-145 microRNA whose overexpression in breast cancer cells reduced the F11R/JAM-A level and decreased the cell migration [19]. This observation prompted the idea of using the F11R/JAM-A inhibitors for clinical purposes, which was supported by later research. Functional approach coupled with proteomic analysis permitted to identify F11R/JAM-A as a promising cancer drug target [20] in conjunction with observations that F11R/JAM-A can serve as a cell surface marker for high-throughput flow cytometry-based characterization of TNBC cancer stem cells (CSCs) [21]. Similarly, the identification of F11R/JAM-A as an essential CSC adhesion protein in patient-derived glioblastoma cells [22] further suggests a carcinogenic role for F11R/JAM-A in driving self-renewal and tumor growth.

Accordingly, different approaches targeting F11R/JAM-A have been tested for anticancer drug therapy. For example, a monoclonal antibody (mAb) against F11R/JAM-A inhibited tumor growth in several mouse models [23]. However, the mAb therapy is expensive and its safety is limited, since high doses of mAb for a prolonged time are needed to obtain the desired efficiency, which further leads to increased immunogenicity caused by high mAb concentrations. Furthermore, the large-scale mAb production from hybridomas is limited by the quantity, quality and the cell line type [23].

Upon inflammation, that is often associated with cancer, F11R/JAM-A molecules are relocated from endothelial TJs to the apical endothelial surface that faces the vessel lumen, where they interact with leukocyte and cancer cells surface proteins, including F11R/JAM-A (by *trans*-homodimerization) and integrins [24]. Thus, the endothelial permeability is increased and F11R/JAM-A actively promotes the adhesion and the subsequent transendothelial migration (TEM) of leukocytes and cancer cells [25–27]. TEM of cancer cells is an initial step of metastasis [26]. Therefore, we have developed the peptide antagonist to F11R/JAM-A, designated as peptide 4D (P4D), that mimics the *trans*-homodimerization interface of F11R/JAM-A molecule, thus binding to this active site and blocking the interactions of endothelium with leukocytes and cancer cells [28]. We have previously shown, that peptide 4D inhibits the early stages of metastasis in breast cancer in vitro [29]. The detailed description of the peptide 4D derived from F11R/JAM-A and scrambled control (Scr) peptide was published previously [28]. Taking into account the reports describing the role of F11R/JAM-A in TNBC [21, 30] and a F11R/JAM-A antagonist Tetrocarcin-A that is proposed as a potential therapeutic compound in TNBC [13] we aimed to test the F11R/JAM-A-derived peptide 4D as a novel potential therapeutic candidate for the treatment of TNBC.

## Material and methods

### In silico analysis

The pan-cancer analysis and the differential gene expression analysis in Tumor, Normal and Metastatic tissues, termed as TNMPlot, are available online [31, 32]. The pan-cancer analysis presented the expression range for F11R gene across all tissues in all available normal (non-cancerous) and tumor RNA (transcriptome) sequencing (RNA-Seq) data. Statistically significant differences by Mann–Whitney U test were marked on the plot with red colour and asterisk. Moreover, the F11R gene expression level was evaluated in breast invasive carcinoma by differential gene expression analysis using RNA-Seq based data from paired tumour and adjacent non-cancerous tissues (n = 112).

The analysis of samples from The Cancer Genome Atlas (TCGA) presenting the expression of F11R/JAM-A based on major subclasses of breast invasive carcinoma (BRCA) was performed using The University of Alabama at Birmingham Cancer data analysis portal (UALCAN) [33–35].

The Kaplan–Meier plot was obtained by the Kaplan–Meier plotter (KM plotter) online survival analysis tool, available at [36] to assess the correlation between the F11R gene expression level and the probability of overall survival (OS) in Triple Negative Breast Cancer (TNBC) [37]. The KM plotter is based upon the information from Gene Expression Omnibus (GEO), European Genome-phenome Archive (EGA), and The Cancer Genome Atlas (TCGA) databases. The KM plotter was performed with the following settings: the probe set was selected by the user with the following Affymetrix ID: 222354_at, the “auto select best cutoff”, “follow up threshols”, “censore at threshold”, “array quality control” options were activated, the cutoff value used in analysis was set to 140, the expression range of the probe was between 8 and 1037, the redundant samples and biased arrays were removed form analysis, the proportional hazards assumption for a Cox regression model using the coxph function was tested with the following result: passed (P = 1), the cohorts were not selected. Using the selected parameters, the analysis was ran on data obtained from 153 patients.

### Cell culture and treatment

The cell lines MDA-MB-231 (ATCC HBT-26, human breast adenocarcinoma, Claudin-low), EA.hy926 (ATCC CRL-2922, derived by fusion of human umbilical vein endothelial cells with continuous human lung carcinoma cell line A549) and HMEC-1 (ATCC CRL-3243, human microvascular endothelial) were purchased from the American Type Culture Collection (ATCC, Manassas, VA, USA). MDA-MB-231 cell line was obtained from a pleural effusion from a breast cancer 51 year old patient in 1973 [38] and represents a commonly used, highly aggressive, invasive and poorly differentiated cell model for TNBC research as it lacks ER and PR expression, and HER2 amplification [39, 40]. MDA-MB-231 cells were cultured in RPMI-1640 medium supplemented with 10% fetal bovine serum (FBS). Human endothelial hybrid cell line EA.hy926 is the best characterized macrovascular EC line [41, 42]. EA.hy926 cells were cultured in Dulbecco’s modified Eagle’s medium (DMEM) with high glucose, supplemented with 10% fetal bovine serum, HAT (100 M hypoxanthine, 0.4 M aminopterin, and 16 M thymidine), and antibiotics in a 90–95% humidified atmosphere of 5% CO_2_ at 37 °C. HMEC-1 cells were grown in MCDB131 medium supplemented with 10 ng/mL epidermal growth factor (EGF), 1 µg/mL hydrocortisone, 10 mM glutamine and 10% FBS. All the cell lines were cultured in a 90–95% humidified incubator of 5% CO_2_ at 37 °C. Confluent cells were passaged using trypsin–EDTA (trypsin-ethylenediaminetetraacetic acid) at a split ratio of 1:2 to 1:4 (MDA-MB-231), 1:4 to 1:5 (EA.hy926), and 1:6 to 1:12 (HMEC-1). The culture media were changed each 2–3 days.

For the inhibition of TJs formation between the cancer cells and endothelium, the cells were left untreated (Control/Ctrl), treated with the peptide 4D (P4D), or with the control peptide (Scr), whose sequence corresponded to P4D peptide, but was scrambled by random insertion of amino acid residues during the chemical synthesis. The peptides were synthesized and purified by LifeTein, LLC and their sequences were as follows: NH2-(dK)-SVT-(dR)-EDTGTYTC-CONH2 for P4D and NH2-S-(dK)-TVE-(dR)-TDTGTYC-OH for Scr. The cells were treated with P4D or Scr (500 µM) or left untreated (Control/Ctrl) for 24 h, unless stated otherwise.

### Cell morphology

Three cell lines: EA.hy926, HMEC-1, MDA-MB-231 were treated with the F11R/JAM-A-derived peptide 4D (P4D) and with the control peptide Scr at a concentration of 500 μM at the time of cell seeding in 6-well plates. The optimal peptide concentration for in vitro experiments was established previously [43]. The cells were incubated for 7 days with the peptides in a cell culture incubator under 5% (v/v) CO_2_ conditions at 37 °C. For the visual estimation of the effect of P4D and Scr peptides on the general cell health, including cell morphology and confluency, the microphotographs in brightfield channel were taken 7 days after seeding using the ZOE Fluorescent Cell Imager (Bio-Rad) in two randomly chosen fields of view of each well.

### Clonogenic assay

EA.hy926, HMEC-1 and MDA-MB-231 cells were seeded at a number of 1 × 10^3^ cells per well and immediately treated with P4D or Scr peptides at 500 μM. The cells were incubated in a humidified incubator under 5% (v/v) CO_2_ conditions at 37 °C. When the colonies were formed (after 14 days), the cells were stained with 0.5% crystal violet in 6% glutaraldehyde for 30 min, washed with water and dried. The plates were placed on a L-130 white transillumination table (Famed, Lodz, Poland), the colonies were macrophotographed with an Olympus C-5050 digital camera and counted with the counter tool of ImageJ software [44]. The plating efficiency (PE) was estimated as follow: (number of colonies formed/number cells seeded) × 100%. The number of colonies that arise after treatment of cells is called the surviving factor (SF) and was calculated as follows:

1 $$\mathrm{SF} = \frac{\mathrm{No}.\mathrm{ of \,colonies \,formed \,after \,treatment }}{\mathrm{No}.\mathrm{ \,of \,cells \,seeded \,}\times \mathrm{PE}}$$

### MTT cell viability assay

The assay was performed by MTT Cell Proliferation Assay Kit (Cayman Item no. 10009365). EA.hy926, HMEC-1 and MDA-MB-231 cells were seeded in culture media at a number of 1 × 10^3^ cells per well in a 96-well plate and immediately incubated for 24 h with P4D or Scr peptides at a concentration of 500 μM in a humidified incubator under 5% (v/v) CO_2_ conditions at 37 °C. Number of replicates for each tested group: n = 16. After 48-h incubation, 10 μl of MTT (3-(4,5-dimethylthiazol-2-yl)-2,5-diphenyltetrazolium bromide) reagent (Cayman Item no. 10009591) was added to the cells followed by further 4-h incubation. To dissolve formazan crystals, 100 μl of crystal dissolving solution was added to each well, followed by further 18 h incubation. The absorbance was read at 595 nm (A_595_) by the Wallac 1420 VICTOR2 Multilabel Counter (PerkinElmer).

### BrdU cell proliferation assay

EA.hy926, HMEC-1 and MDA-MB-231 cells were seeded in culture media at a density of 1 × 10^4^ cells/well in a 96-well plate and immediately incubated for 24 h with P4D or Scr peptides at a concentration of 500 μM in a humidified incubator under 5% (v/v) CO_2_ conditions at 37 °C. Number of replicates for each tested group: n = 16. After 48h incubation, 10 μM BrdU (5-bromo-2ʹ-deoxyuridine) was added to the cells followed by further 24 h incubation. BrdU incorporation into newly synthesized DNA of actively proliferating cells was detected by ELISA (enzyme-linked immunosorbent assay) with BrdU Cell Proliferation Assay Kit (Cell Signaling, #6813) according to the manufacturer’s protocol. The absorbance was read at 450 nm (A_450_) by the Wallac 1420 VICTOR2 Multilabel Counter (PerkinElmer).

### Transepithelial/endothelial electrical resistance (TEER) measurements

The cell monolayers were cultured on 24-well Tissue Culture Plate Inserts with the polycarbonate semipermeable membrane of 0.4 μm pores and 6.5 mm diameter (VWR International, LLC, Radnor, PA, USA) with P4D or Scr peptides at a concentration of 500 μM in a humidified incubator under 5% (v/v) CO_2_ conditions at 37 °C for 24 h. Resistance (R) measurements were performed by EVOM3 instrument equipped with EndOhm chamber (World Precision Instruments, Sarasota, FL, USA) due to the manufacturer’s protocol and their results were expressed in ohms (Ω). TEER was calculated as follows:

$$\mathrm{TEER }[\Omega \times {\mathrm{cm}}^{2}] =\mathrm{ R }[\Omega ] \times \mathrm{ EMA }[{\mathrm{cm}}^{2}]$$

where EMA is the Effective Membrane Area, that is the area of semipermeable polycarbonate membrane available for the cell culture, and for 24-well inserts its value equals 0.3316 cm^2^. TEER values are expressed in Ω × cm^2^.

### Mice mammary gland tumor culture and tumor induction

Female BALB/cAnNCrl mice aged 8 weeks from the Charles River Laboratories were housed under standard conditions at the Animal research facility and in vitro testing laboratory of the Medical University of Lodz (Lodz, Poland), with a management system implementation according to PN-EN ISO/ EC 17025:2005. The procedures were conducted as per the regulations of the National Ethics Committee for Animal Experiments operating at the Ministry of Science and Higher Education of Poland, and were approved by the Local Ethical Committee for Animal Research in Lodz, Poland (license number 13/LB 164/2020). All efforts were made to minimize animal suffering, compatibly with EU/2010/63 Directive and the amendment of the law on the protection of animals used for scientific or educational purpose. Murine 4T1 cells (ATCC) were cultured in RPMI-1640 with 10% FBS in flasks to 80% confluence. The 4T1 cell line is widely used for the induction of metastatic triple-negative breast cancer mouse model [45, 46]. The mouse model of TNBC was developed by the orthotopic injection of viable 4T1 cancer metastatic cells into mammary fat pads in all mice (twenty-four individuals). Briefly, the 4T1 cells were harvested, resuspended at a concentration of 1 × 10^4^ cells in 100 µl PBS (phosphate buffered saline) per mouse, and administered subcutaneously in inguinal nipple area. All injections were performed on the same day. 7 days after the injections, the mice were divided into three groups: two P4D groups (P4D injections, group no.1 and group no. 2), or the control group (vehicle injections, group no. 3). The mice of both P4D groups were treated by the intraperitoneal daily injections of P4D resuspended in 0.9% saline at a concentration of 200 mg/1 kg body weight/mouse (4 mg of P4D in 200 µl of 0.9% saline per mouse for group no. 1 and 0.4 mg of P4D in 200 µl of 0.9% saline per mouse for group no. 2). The control group was treated with an equivalent amount of diluent vehicle (200 µl of 0.9% saline per mouse for group no. 3). Eight mice were used for each experimental group. Allocation to experimental groups was based on simple randomization. During the experiments, the animals were housed under standard conditions and constant veterinary supervision, with free access to water and standard chow for rodents. The animals were under constant veterinary supervision: routine veterinary inspections assessing the overall welfare and the estimation of body mass of the animals were carried out daily. Each day the animals were manually checked for the tumour presence by palpation test. After 21 days of treatment, the mice were anesthetized intraperitoneally with the ketamine and xylazine solution (0.1 ml/20 g mouse) and weighed. The murine spleens, livers, lungs, and primary tumours were dissected, and the spleens and primary tumours were weighed. The livers and lungs were fixed 10% buffered formalin for at least 72 h before the histopathological evaluation of metastases in 4T1 mouse model of breast cancer. Blood samples were collected by cardiac blood drawn using a syringe with K2-EDTA solution. Samples were centrifuged (662 ×*g*, 10 min, 4 °C), and the plasma was aliquoted and stored at − 80 °C for further analysis.

### Measurements of F11R/JAM-A antigen levels in murine blood plasma by sandwich enzyme-linked immunosorbent assay (ELISA)

F11R/JAM-A levels in murine plasma were evaluated by RayBio Mouse JAMA ELISA Kit (RayBiotech, cat.# ELM-JAMA) according to manufacturer’s protocol. For the sandwich ELISA assay murine plasma was diluted 5 times with an assay diluent, provided by the manufacturer with the kit. The samples from each of the three experimental groups (n = 8) were assayed in triplicates. Spectrophotometrical measurements of absorbance were performed by the Wallac 1420 VICTOR2 Multilabel Counter (PerkinElmer) at 450 nm (A_450_).

### Histopathological evaluation of metastasis in a mouse model of breast cancer

Breast cancer metastasis in the lungs and liver were investigated by routine histopathological examination after fixation of specimens in 10% buffered formalin for 48 h. The tissue samples were embedded in paraffin (FFPE–formalin-fixed, paraffin-embedded blocks), and 5 μm-thick sections were stained with hematoxylin and eosin, or with specific antibodies. For immunohistochemistry staining the following antibodies were used: anti-mouse JAM-A polyclonal goat IgG (R&D; #AF1077), and anti-mouse HER2/ErbB2 (29D8) Rabbit mAb (Cell Signaling Technology; #2165S). Both antibodies display cross reactivity with mice according to manufacturer’s and literature data [47, 48]. The histological appearance of the tissue was examined by light microscopy with the use of an inverted microscope with a standard colour camera (Axiolab 5 with Axiocam 208 Color, Zeiss). The lung metastases were preliminary evaluated in three following sections under magnifications of 40 × , 100 × and 400 × . Subsequently, the size of each section was measured, the metastases were quantified and the largest dimension of metastatic foci for each section was measured. The detailed morphological analysis of metastatic foci was conducted in digitalized images using an UltraFast Scanner (Philips IntelliSite Solution, USA) with DigiPath^™^ Professional Production Software (Xerox, Norwalk, CT, USA).

### Statistical analysis

The results are presented as arithmetic mean ± standard deviation (SD) of at last three independent experiments. The data were statistically analyzed and plotted for the graphical presentation by the GraphPad Prism v. 6.01 statistical software (GraphPad Software, Inc.). An assessment of the normality of data was performed by Shapiro–Wilk W test with Dell Statistica v. 13.1 data analysis software system (Dell, Inc.). Consequently, the data falling within Gaussian distribution were analyzed with the GraphPad Prism software by ordinary one-way analysis of variance (ANOVA) with the Tukey’s post hoc multiple comparisons test. Otherwise, the non-parametric Kruskal–Wallis test followed by Dunn’s multiple comparison test was applied. Differences were considered statistically relevant at P < 0.05. The degree of association between the mass of spleen and primary tumours obtained from mice was tested with Spearman’s rank correlation.

A more thorough statistical analysis was performed for the data obtained from in vivo experiments on 4T1 breast cancer murine model. Due to small sample sizes (n = 8) and the resulting low statistical power of the estimated interferences, the resampling bootstrap technique with 10^6^ iterations and with the assumption, that the tested groups are three times greater (predicted group size n = 24) was used with the implementation of the Resampling Stats for Excel v. 4.0 add-on to determine the likelihood of obtaining the revealed differences due to pure chance, followed with Kruskal–Wallis test and with the Conover-Iman all pairwise comparisons *post-hoc* test. The post hoc statistical power to estimate the minimum sample size that offers a sufficient statistical power was calculated by Kruskal–Wallis test using the Study Size software (version 3.00, Bertil Olofsson, CreoStat HB, Enbarsv.11, 426 55 V. Frolunda, Sweden).

## Results

### In silico analysis of F11R gene expression in breast cancer

**Fig. 1:**
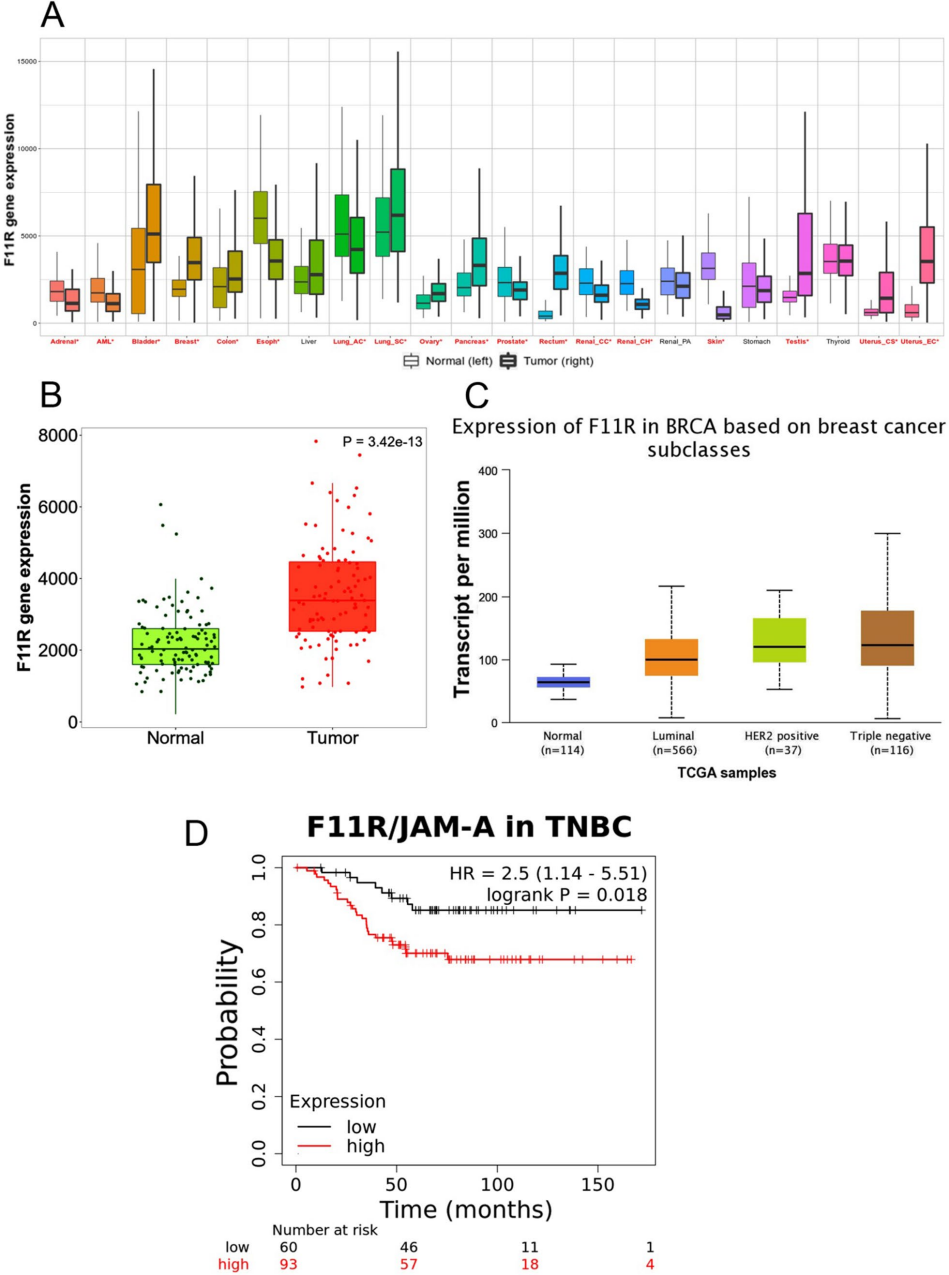
The results of in silico analyses. (**A**) The pan-cancer analysis performed by the web tool TNMplot (https://tnmplot.com/analysis/), showing the expression range for F11R gene across all tissues in all available normal (non-cancerous) and tumor RNA-Seq data. Statistically significant differences by Mann–Whitney U test are marked with red colour and asterisk. *AML* acute myeloid leukemia, *Lung_AC* lung adenocarcinoma, *Lung_SC* lung squamous cell carcinoma, *Renal_CC* renal clear cell carcinoma, *Renal_CH* renal chromophobe cell carcinoma, *Renal_PA* renal papillary cell carcinoma, *Uterus_CS* uterine carcinosarcoma, *Uterus_EC* uterine corpus endometrial carcinoma. **B** Differential F11R gene expression analysis in breast invasive carcinoma performed by TNMplot using RNA-Seq based data from normal and tumour tissues, including paired tumour and adjacent non-cancerous tissues (n = 112). **C**. The UALCAN (The University of Alabama at Birmingham Cancer data analysis Portal) Resource analysis on samples from The Cancer Genome Atlas (TCGA), presenting the expression of F11R/JAM-A in breast cancer major subclasses of breast invasive carcinoma (BRCA). **D** Kaplan–Meier plot for F11R gene expression in Triple Negative Breast Cancer (TNBC) based on Gene Expression Omnibus (GEO), European Genome-phenome Archive (EGA), and The Cancer Genome Atlas (TCGA) databases to assess the overall survival (OS) probability. P = 0.0184

The pan-cancer analysis performed by a TNMPlot online tool (https://tnmplot.com/analysis/) of the available normal (non-cancerous) and tumor RNA-Seq data shows that the level of F11R gene product was significantly altered (increased or decreased) in virtually all cancer types, excluding renal papillary cell carcinoma, liver, stomach and thyroid cancer (Fig. 1A). The most pronounced increase of F11R expression was noticed for breast, testis, and uterus cancer. Figure 1B shows the F11R gene expression analysis in breast invasive carcinoma using normal and tumour RNA-Seq based data, including paired tumour and adjacent non-cancerous tissues. The expression level of F11R gene was distinctly elevated in tumour tissues. The UALCAN analysis of TCGA samples performed to compare F11R gene level in ER/PR/HER2 subtypes of BC presented in Fig. 1C has revealed, that F11R expression was abundant particularly in TNBC. The Kaplan–Meier plot in Fig. 1D shows that the increased F11R gene expression level correlated with decreased probability of overall survival (OS) in TNBC (P = 0.0184), indicating therefore the poor prognosis. In a summary, the overexpression of F11R gene is characteristic for breast cancer–that is particularly prevalent in TNBC–and linked with poor prognosis, that is related with the decreased survival rate.

### In vitro study on human endothelial cell lines and a triple negative breast cancer cell line

In our previous report we have demonstrated, that P4D inhibited the interactions between breast cancer and endothelial cells, namely adhesion and transendothelial migration, that are the early stages of metastasis [29]. In this study we focused on the effect of P4D separately on TNBC cells and on endothelial cells.

**Fig. 2:**
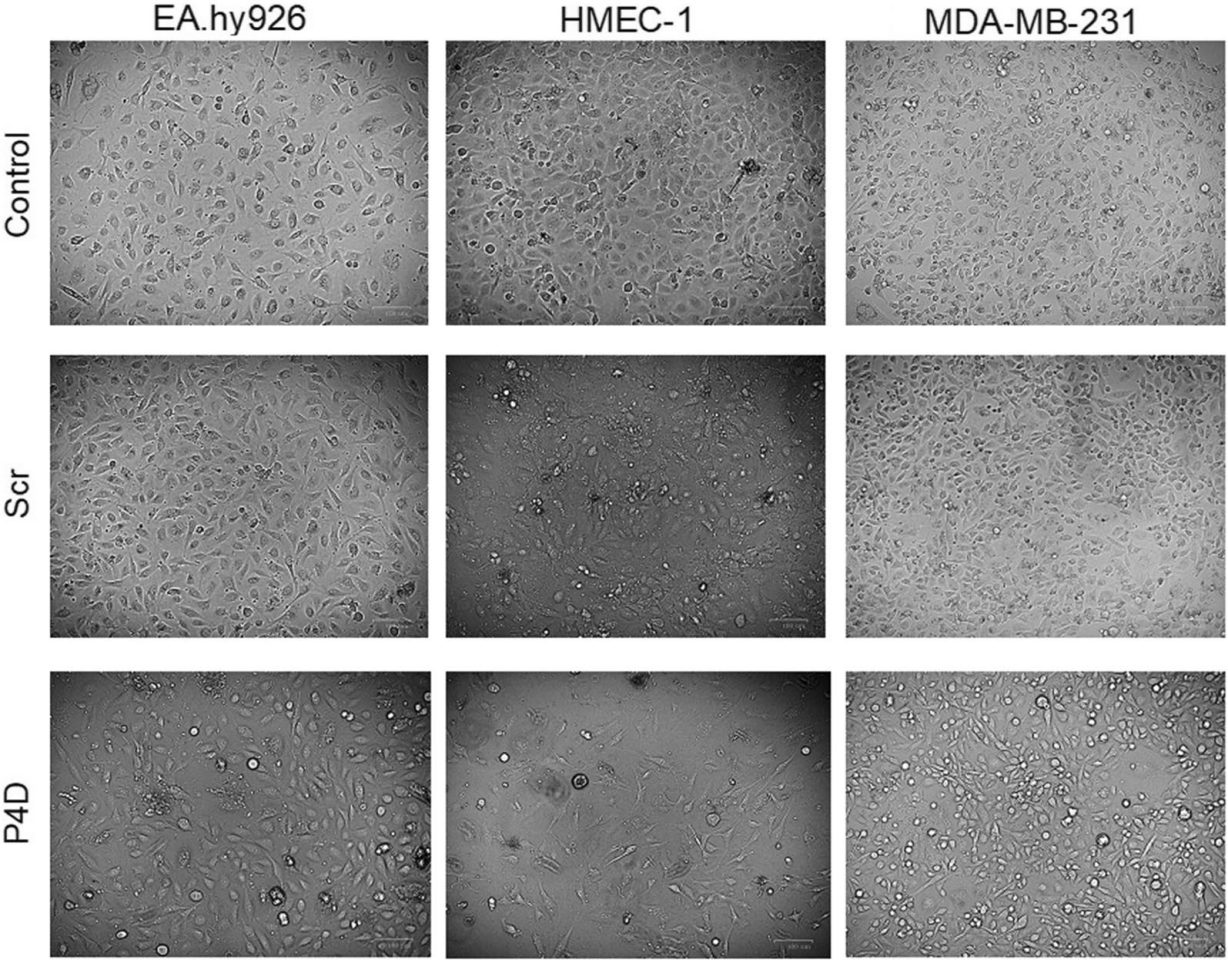
Effect of the F11R/JAM-A-derived peptide 4D on cell morphology. The EA.hy926, HMEC-1, and MDA-MB-231 cells were untreated (Control), treated with the control scrambled peptide (Scr), or with the F11R/JAM-A antagonistic peptide (P4D) at a concentration of 500 μM at the time of cell seeding in 6-well plates. Seven days after seeding the microphotographs in brightfield channel were taken using the ZOE Fluorescent Cell Imager (Bio-Rad) in two randomly chosen fields of view of each well

The F11R/JAM-A antagonistic peptide P4D has altered the morphology of the endothelial (EA.hy926, HMEC-1) and breast cancer (MDA-MB-231) cell lines grown for 7 days in monolayers (Fig. 2). The endothelial cells EA.hy926 and HMEC-1 were more elongated and less confluent when incubated with P4D as compared with the untreated cells (Control) or with the cells incubated with the control peptide (Scr). Moreover, the numerous protrusions were observed in HMEC-1 cells due to decreased confluency. Breast cancer cells of MDA-MB-231 line were also more elongated after incubation with P4D, but the protrusion were not as numerous as in the case of HMEC-1 cells, since the confluency was barely decreased. The interesting feature observed for the P4D-treated cells was the presence of cell-free areas in cell monolayer, but in the case of MDA-MB-231 cells they were less significant as compared with those of endothelial cells (Fig. 2).

**Fig. 3:**
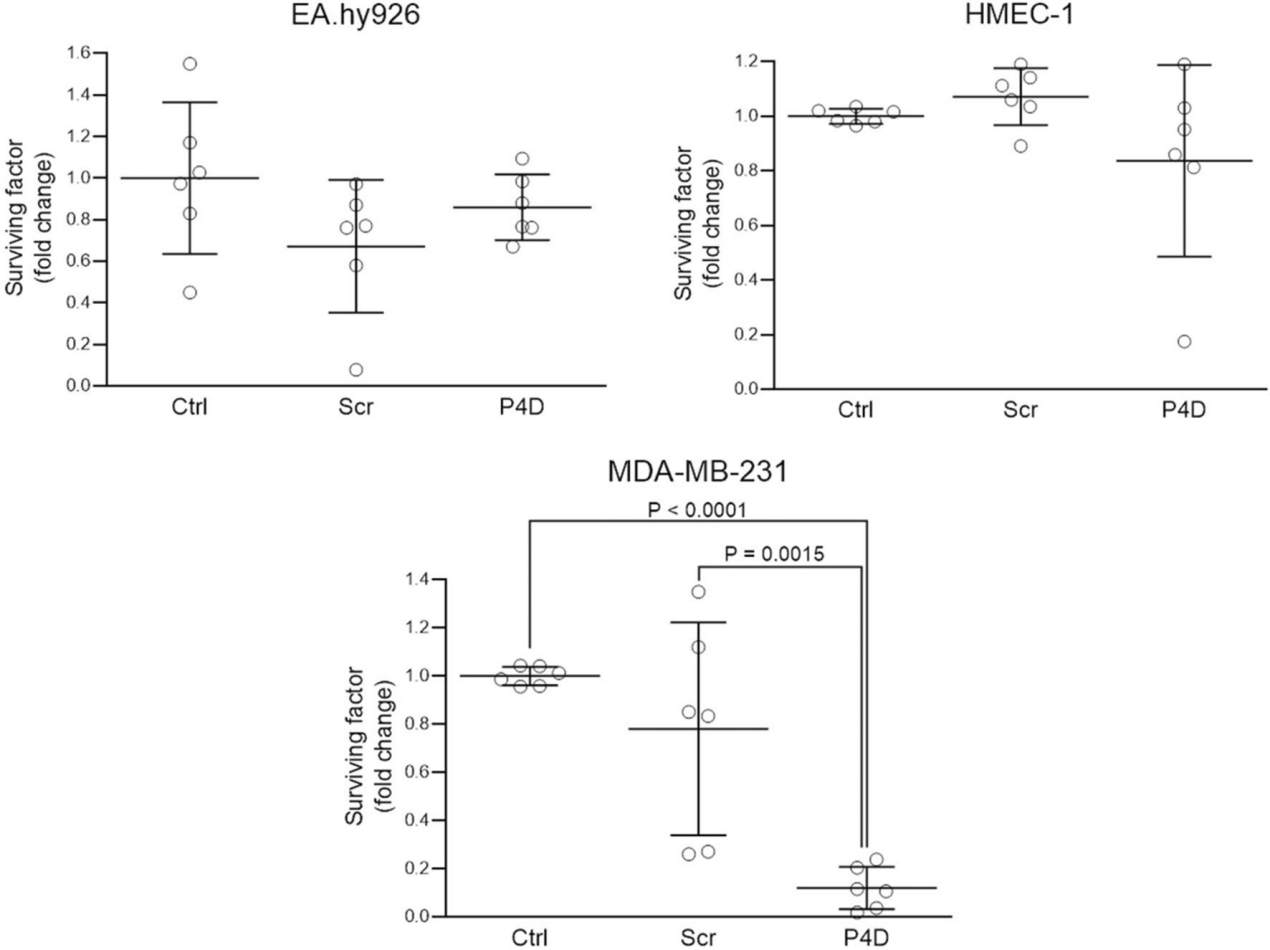
Effect of F11R/JAM-A-derived peptide (P4D) on colony formation by endothelial and breast cancer cells. The colonies were counted 14 days after cell incubation with the peptides and were expressed as the surviving factor (SF), that is the number of colonies that arise after treatment of cells. SF was calculated as described in Materials and Methods section. The cells were untreated (Ctrl), treated with the control scrambled peptide (Scr) or with the F11R/JAM-A antagonistic peptide (P4D) at a concentration of 500 μM. Shapiro–Wilk normality W test was performed whether the data fall upon Gaussian distribution: EA.hy926–passed (P-values for Ctrl: 0.9591, for Scr: 0.1546, for P4D: 0.7538); HMEC-1–passed (P-values for Ctrl: 0.5587, for Scr: 0.6681, for P4D: 0.1515); MDA-MB-231–passed (P-values for Ctrl: 0.2779, for Scr: 0.4005, for P4D: 0.5815). Subsequently, the statistical analysis was performed by one-way ANOVA followed, where applicable, by Tukey’s multiple comparison test. The differences between the groups where found to be not significantly different for EA.hy926 (P = 0.1863) and for HMEC-1 (P = 0.1774). The differences between the MDA-MB-231 groups were significant (P < 0.0001) and the results of Tukey’s multiple comparisons test were as follows: for Ctrl vs. Scr P = 0.3410; for Ctrl vs. P4D P < 0.0001; for Scr vs. P4D P = 0.0015

Peptide 4D also inhibited the ability for continued proliferation of breast cancer cell line MDA-MB-231 in a statistically significant manner, whereas the colony forming capacity of endothelial cell lines was not affected, as estimated by colony forming assay (CFA; Fig. 3).

**Fig. 4:**
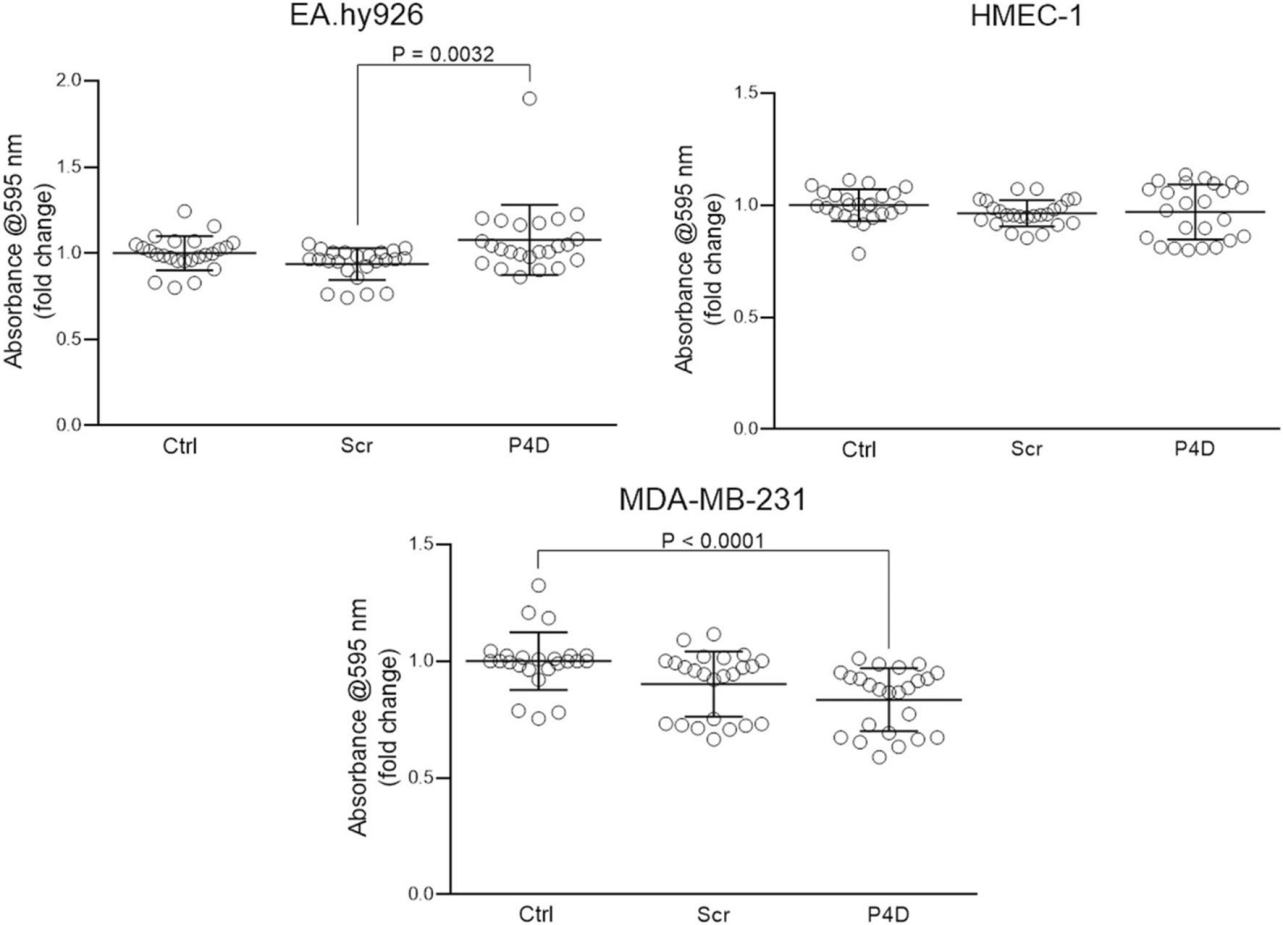
Effect of F11R/JAM-A-derived peptide (P4D) on cell viability measured by MTT assay. The cells were untreated (Ctrl), treated with the control scrambled peptide (Scr) or with the F11R/JAM-A antagonistic peptide (P4D) at a concentration of 500 μM. Shapiro–Wilk normality W test has shown that the data do not fall upon Gaussian distribution: EA.hy926–failed (P-values for Ctrl: 0.3657, for Scr: 0.0009, for P4D: < 0.0001); HMEC-1–failed (P-values for Ctrl: 0.1067, for Scr: 0.6482, for P4D: 0.0072); for MDA-MB-231–failed (P-values for Ctrl: 0.0022, for Scr: 0.0042, for P4D: 0.0113). Thus, the data were analyzed by non-parametric Kruskal–Wallis test followed, where applicable, by Dunn’s multiple comparisons test. The differences between tested groups were statistically significant in the case of EA.hy926 cells (P = 0.0041). The post-hoc test has not revealed significant differences for Ctrl vs. Scr (P = 0.1091) and for Ctrl vs. P4D P = 0.7106. The difference was significant for Scr vs. P4D (P = 0.0032). For HMEC-1 cells the differences between the tested groups were statistically insignificant (P = 0.2281). The statistically significant differences between the tested groups were noted for MDA-MB-231 cells (P < 0.0001). The post-hoc Dunn’s multiple comparisons test has shown, that the differences CTRL vs. Scr and Scr vs. P4D were not statistically significant (P = 0.0526; and P = 0.1002; respectively), while the significant difference was found for Ctrl vs. P4D (P < 0.0001)

The cell viability was significantly decreased by P4D for MDA-MB-231 breast cancer cell line only as measured by MTT assay (Fig. 4, CTRL vs. P4D: P < 0.0001); the mean fold change of A_595_ was at the level of 0.83 ± 0.13 (mean ± SD) as compared with control cells (CTRL). The slight, but statistically significant (P = 0.0032) increase in viability upon P4D stimulation of EA.hy926 cells was the effect of the artefact: an unusually high increase of absorbance in the case of one particular data point with a fold change of 1.90, while an average fold change ± SD = 1.10 ± 0.20.

**Fig. 5:**
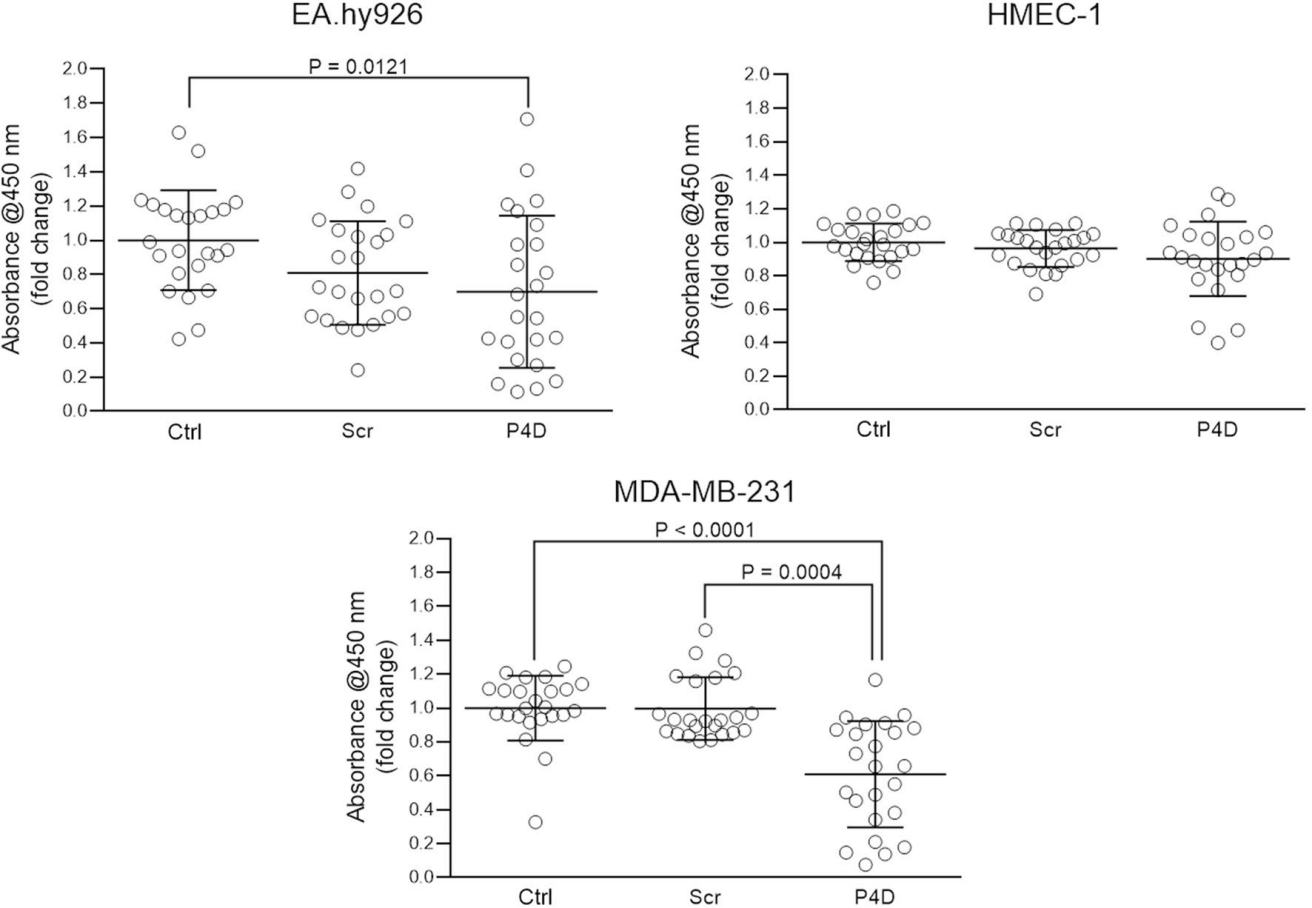
Effect of F11R/JAM-A-derived peptide (P4D) on cell proliferation evaluated by BrdU incorporation assay. The cells were untreated (Ctrl), treated with the control scrambled peptide (Scr) or with the F11R/JAM-A antagonistic peptide (P4D) at a concentration of 500 μM. The data were tested for normality by Shapiro–Wilk W test: EA.hy926 – passed (P-values for Ctrl: 0.6137, for Scr: 0.4433, for P4D: 0.2206); HMEC-1 – passed (P-values for Ctrl: 0.9044, for Scr: 0.3065, for P4D: 0.2034); MDA-MB-231 – failed (P-values for Ctrl: 0.0009, for Scr: 0.0013, for P4D: 0.1616). The data falling into the Gaussian distribution were statistically analyzed by ANOVA (P = 0.0419 for EA.hy926: the differences between the tested groups are statistically significant; P = 0.0956 for HMEC-1: no statistically significant differences) followed, where applicable, by Tukey's multiple comparisons test. Otherwise, the results were analyzed by Kruskal–Wallis test (P < 0.0001 for MDA-MB-231: significant differences between the experimental groups) followed by Dunn's multiple comparisons test. Tukey’s multiple comparisons test for EA.hy926: Ctrl vs. Scr P = 0.1542; Ctrl vs. P4D P = 0.0121; Scr vs. P4D P = 0.5348. Dunn’s multiple comparisons test for MDA-MB-231: Ctrl vs. Scr P = 0.8186; Ctrl vs. P4D P < 0.0001; Scr vs. P4D P = 0.0004

Likewise, P4D significantly impeded the proliferation of MDA-MB-231 TNBC cell line (CTRL vs. P4D: P < 0.0001; Scr vs. P4D: P = 0.0004), whereas the proliferation of HMEC-1 endothelial cell line was not disturbed (Fig. 5). We have also noted the slight, but still statistically significant decrease of EA.hy926 proliferation, while comparing the difference in proliferation rate between the CTRL and P4D group (mean fold change ± SD = 0.70 ± 0.44; P = 0.0121).

**Fig. 6:**
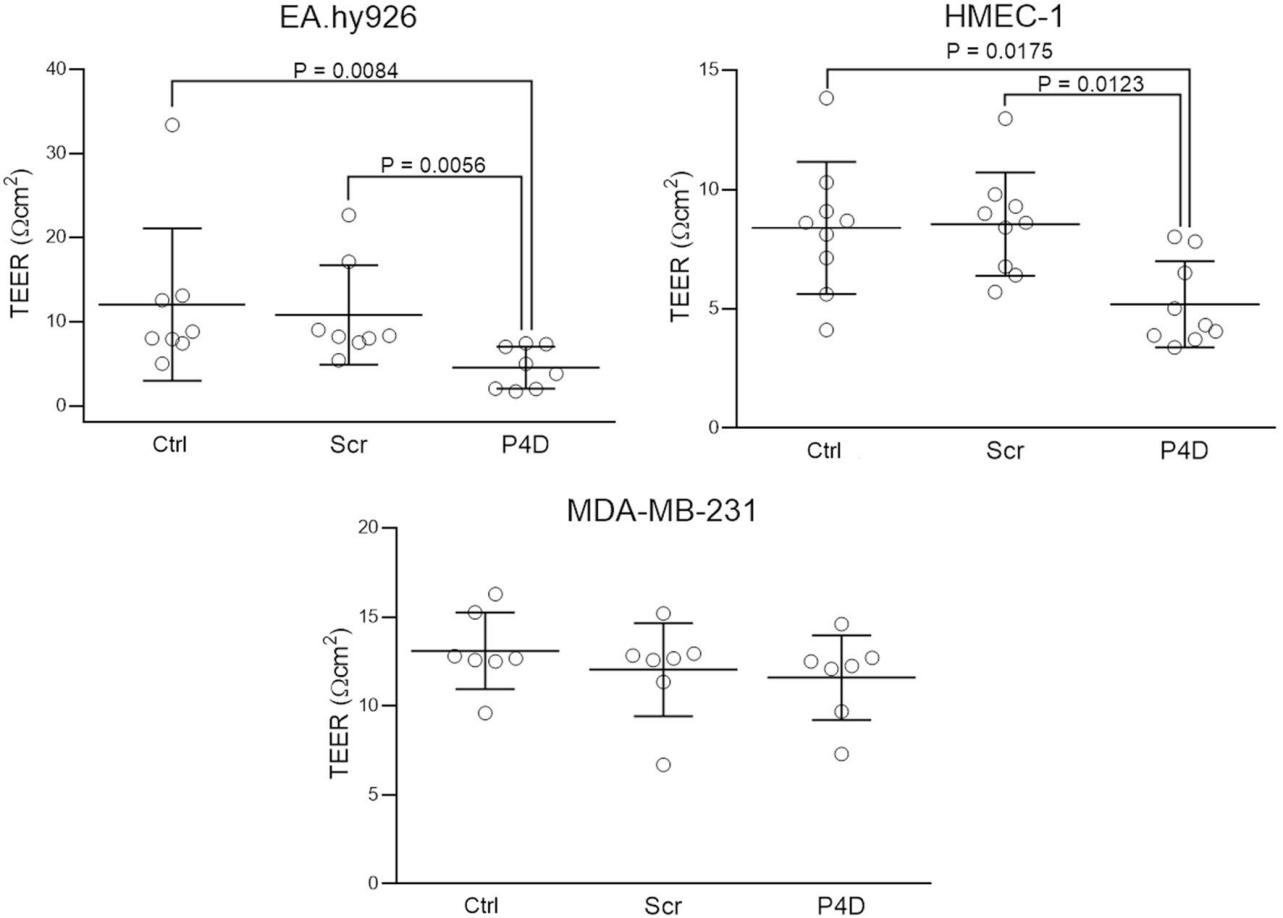
Effect of F11R/JAM-A-derived peptide (P4D) on the cell monolayer permeability measured by EVOM3 instrument. The cells were untreated (Ctrl), treated with the control scrambled peptide (Scr) or with the F11R/JAM-A antagonistic peptide (P4D) at a concentration of 500 μM. The monolayer permeability is inversely proportional to TEER (trans epithelial/endothelial electrical resistance) values, specified in Ω × cm^2^. The data were tested for normality by Shapiro–Wilk W test: EA.hy926–failed (P-values for Ctrl: 0.0016, for Scr: 0.0114, for P4D: 0.0875); HMEC-1–passed (P-values for Ctrl: 0.7985, for Scr: 0.5107, for P4D: 0.0630); for MDA-MB-231–passed (P-values for Ctrl: 0.3698, for Scr: 0.0679, for P4D: 0.3424). The permeability results obtained with EA.hy926 cells were analyzed by Kruskal–Wallis test: P = 0.0020–the mean TEER values are significantly different. Dunn’s multiple comparisons test of data obtained on EA.hy926 cells revealed, that permeability of P4D group differs significantly from control groups: Ctrl vs. Scr P > 0.9999; Ctrl vs. P4D P = 0.0084; Scr vs. P4D P = 0.0056. The results of HMEC-1 and MDA-MB-231 monolayer permeability measurements were tested by ordinary one-way ANOVA. For HMEC-1 cells the differences between the groups were statistically significant: P = 0.0067. As evidenced by Tukey’s multiple comparisons test for data derived on HMEC-1 cells, the permeability of P4D group differs significantly from control groups: Ctrl vs. Scr P = 0.9876; Ctrl vs. P4D P = 0.0175; Scr vs. P4D P = 0.0123. For MDA-MB-231 cells the differences between the groups were missing the statistical significance: P = 0.4909

The effect of F11R/JAM-A-derived peptide on cell monolayer integrity was estimated by TEER measurements. As presented in Fig. 6, the P4D antagonistic peptide increased the permeability of endothelial cell monolayers that was manifested by the significantly decreased TEER values for P4D-treated monolayers as compared with non-treated (CTRL) and Scr-treated cells, while the epithelial monolayer permeability of TNBC MDA-MB-231 cells was not affected by P4D.

Moreover, we have performed the scratch wound assay to compare the migratory potential of the endothelial and TNBC cells upon P4D stimulation. The cells were grown in 6-well plates and the scratch was made in each well with a 200 μl sterile pipette tip to form a gap that induces the cells to migrate and close the gap, thus the one-directional cell migration was analysed by this technique. However, we did not observe any effect of P4D on one-directional migratory potential of the cells. The detailed protocol and the plots presenting the results of scratch assay can be found in the Additional file 1: (Additional file materials and Methods and Additional file 1: Figure S1).

Taken together, the results of our in vitro experiments show that the F11R/JAM-A antagonistic peptide P4D has reduced the viability and inhibited the growth of MDA-MB-231 TNBC cells without affecting the functions of endothelial cell lines EA.hy926 and HMEC-1. Simultaneously, the confluency of endothelial cell monolayers was loosened upon P4D treatment, whereas the TNBC cell monolayer integrity was not altered. These observations were supported by the morphological changes exerted on the endothelial and TNBC cells by P4D, as presented on brightfield microphotographs.

### Experimental anticancer therapy with F11R/JAM-A antagonistic peptide P4D in 4T1 murine triple negative breast cancer model

We have previously demonstrated, that the F11R/JAM-A antagonistic peptide 4D blocks the interactions between breast cancer cells and endothelial monolayer, including adhesion and TEM in vitro [29]. The current report further confirms the previously published observations showing, that P4D decreases the viability and proliferation of TNBC cells, while disturbing the cellular interactions within the endothelial monolayer, thus increasing its permeability. To verify the relevance of these findings to in vivo conditions, the experimental anticancer therapy with F11R/JAM-A peptide was performed on the mouse 4T1 breast cancer model. Primary tumors were induced by inoculation the mice with the 4T1 breast cancer cells subcutaneously in the mammary gland. Seven days later the mice were divided in 3 experimental groups of 8 mice in each group and the following daily intraperitoneal injections were administered for 21 days:

 − Group no. 1: 4 mg of P4D in 200 µl of vehicle per mouse (‘P4D 4.0 mg’ group);
 − Group no. 2: 0.4 mg of P4D in 200 µl of vehicle per mouse (‘P4D 0.4 mg’ group);
 − Group no. 3: 200 µl of vehicle (0.9% sodium chloride) per mouse (‘Control’ group).

After the injections, the mice were euthanized, followed by blood, spleen, primary tumors, liver, and lung collection. The blood was centrifuged and the plasma was used for ELISA analysis. The primary tumors and spleen specimens were weighed, while the lungs and livers were subjected to histopathological analysis for the presence of metastatic lesions. The mean body mass of the mice in each experimental group one day before the treatment was as follows (mean value in grams ± SD): 20 ± 1.5 g for Control; 19 ± 1.6 g for P4D 0.4 mg; 20 ± 1.2 g for P4D 4.0 mg. The mean body mass of the mice in each experimental group after the euthanasia was 21 ± 1.9 g for Control; 19 ± 1.2 for P4D 0.4 mg; and 20 ± 1.3 for P4D 4.0 mg, thus the average body mass of the mice did not change significantly in each experimental group during the experiment. Likewise, the mean mass of primary tumours and spleen specimens dissected from mice did not differ significantly between the experimental groups (Additional file 2). We have found a clear correlation between the mass of primary tumour and spleen for Control group (Spearman: r = 0.8333) and for P4D 4.0 mg group (Spearman: r = 0.8095): both quantities are directly proportional one to another, while the correlation for P4D 0.4 mg group was not significant (Spearman: r = 0.1905). The Spearman plots showing the correlation between the mass of primary tumours and spleen for all experimental groups are presented in Additional file 1: Figure S2. The raw data of the daily routine inspections of 4T1 mice and the morphological analysis after euthanasia are presented in Additional file 2, while the tables presenting the raw data of histopathological analysis can be found in (Additional file 1: Tables S1, S2, and S3).

**Fig. 7:**
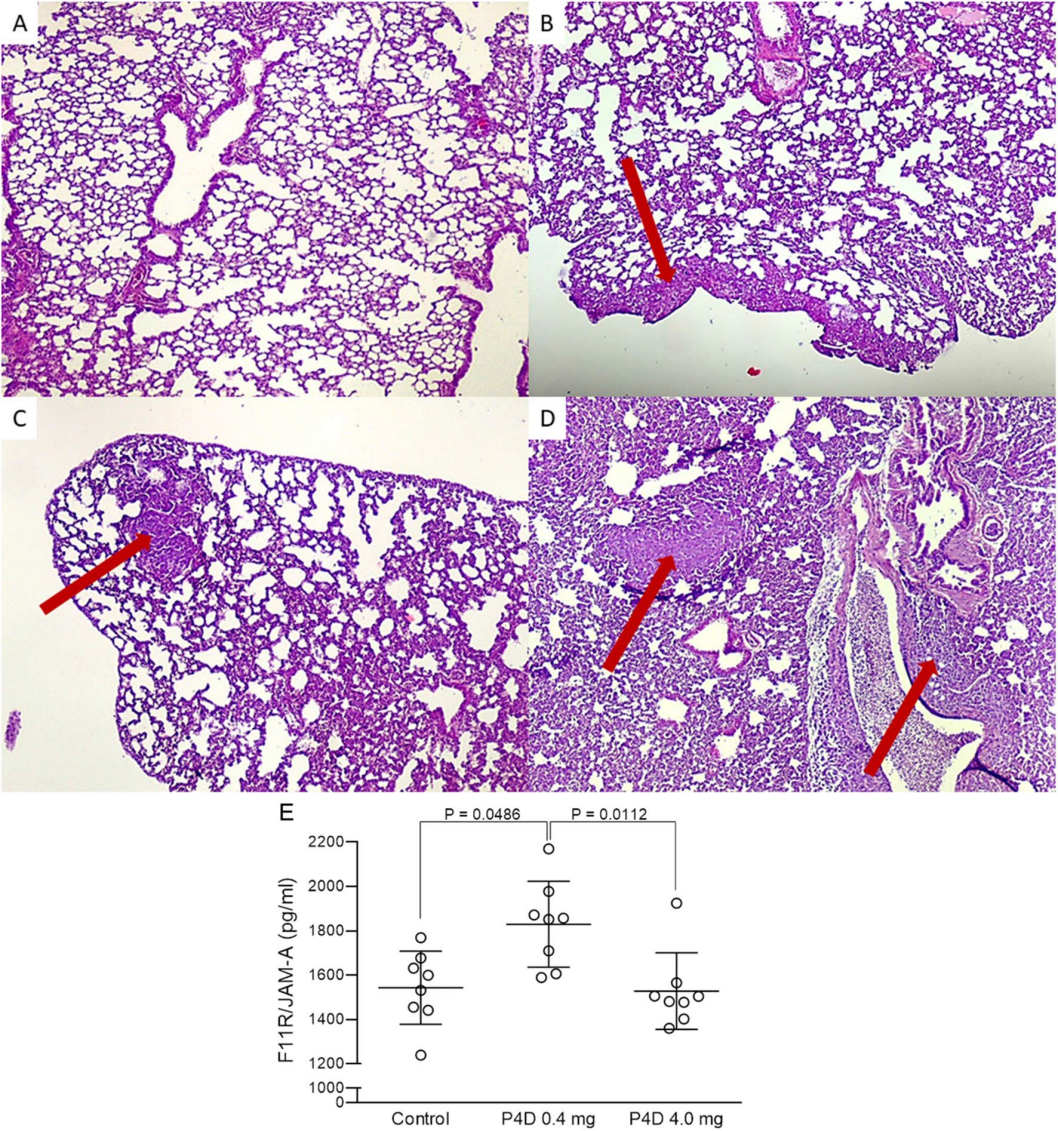
Effect of F11R/JAM-A-derived peptide (P4D) on metastasis in 4T1 mouse breast cancer model. The peptide P4D decreases the breast cancer metastasis to the lungs in mice. The mice were treated for 21 days with daily injections of 4 mg P4D (group 1), 0.4 mg P4D (group 2) or vehicle (0.9% saline, group 3). Representative hematoxylin and eosin stained images of metastases in the 4T1 mouse breast cancer model are shown. **A**: lung parenchyma without metastases. **B**–**C**: Lung parenchyma with small metastases (**B**: group 1, **C**: group 2). **D**: Massive metastases to the lung (group 3). Metastases are indicated by red arrows. Magnification of 400 × . **E**: F11R/JAM-A antigen level in blood plasma from 4T1 control mice and mice treated with two concentrations of peptide 4D. The data do not fall upon Gaussian distribution as tested by Shapiro–Wilk normality W test (P-values for Control: 0.8942, for P4D 0.4 mg: 0.6287, for P4D 4.0 mg: 0.0119). As estimated by Kruskall-Wallis test (P = 0.0081) the mean values of F11R/JAM-A level in murine plasma different significantly between the groups. Dunn’s multiple comparisons test revealed, that the significant differences are found for P4D 0.4 mg vs. Control (P = 0.0486), and for P4D 0.4 mg vs. P4D 4.0 mg (P = 0.0112). For Control vs. P4D 4.0 mg the difference was not within the statistical significance (P = 1.0000)

The histopathological analysis of the murine lungs has revealed evident anti-metastatic effect of P4D (Fig. 7A–D). We have observed large and numerous metastases in the lung parenchyma of the control mice (Fig. 7D) and several small metastases in the lung parenchyma of the mice treated with 0.4 mg injections of P4D (Fig. 7C) and a few small or even no metastases in the lung tissue specimens from mice treated with 4.0 mg of P4D (Fig. 7A, B). Figure 7E shows the results of ELISA analysis of the F11R/JAM-A antigen level in the murine blood plasma. We have noted the statistically significant difference between the mean value of F11R/JAM-A level from mice treated with the 0.4-mg injections and with that one from control mice (P = 0.0486), and from mice treated with 4.0-mg P4D injections (P = 0.0112). This result is quite unexpected and the most probably is the effect of an artefact.

**Fig. 8:**
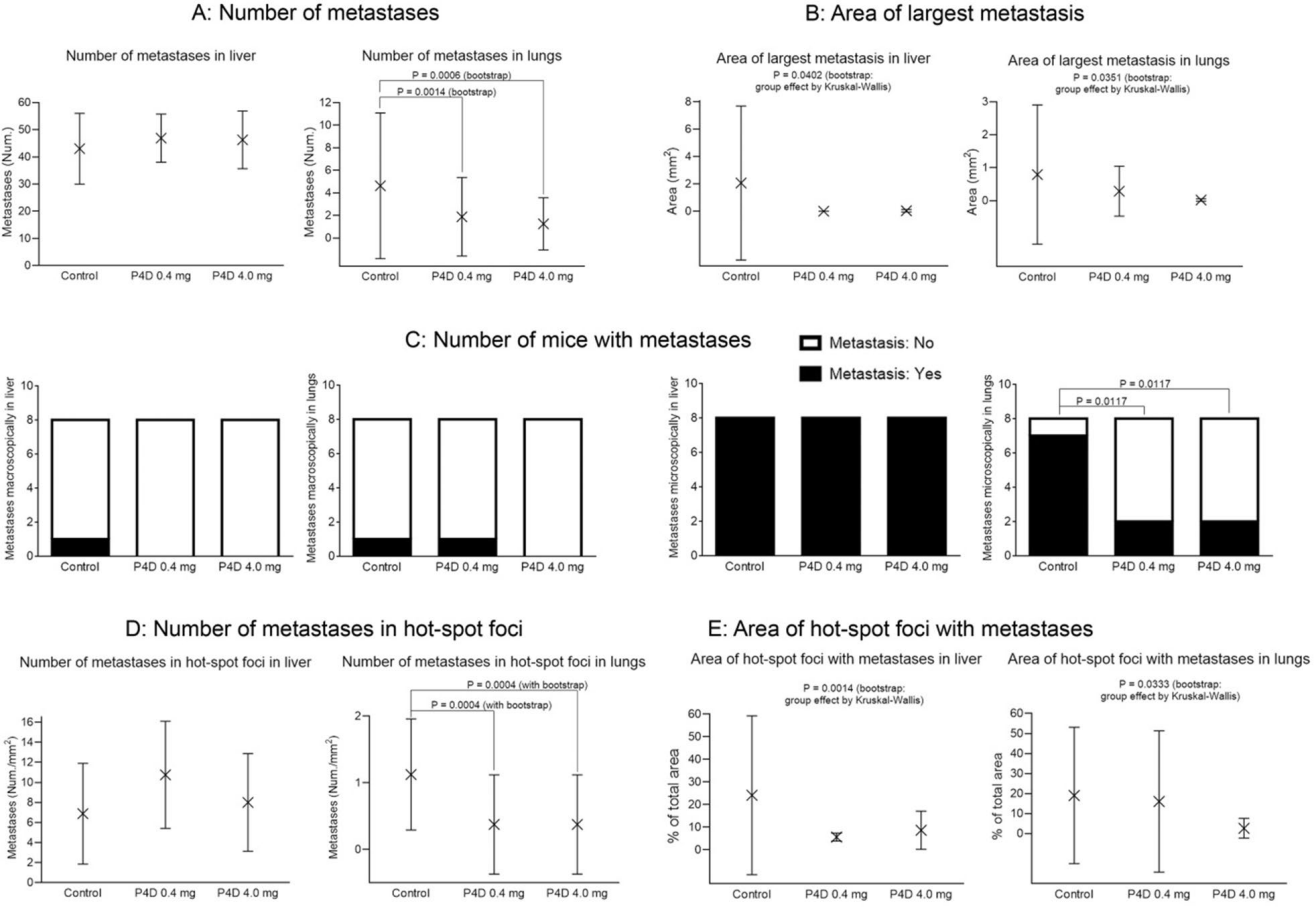
The F11R/JAM-A antagonistic peptide (P4D) impairs the metastasis in 4T1 mouse breast cancer model. **A**: Effect of P4D peptide on number of metastases in liver and lungs from 4T1 mice. **B**: Area of largest metastasis in liver and lungs from control 4T1 mice and mice treated with two concentrations of P4D. **C**: Effect of P4D on number of mice with metastases observed macroscopically and microscopically in liver and in lungs. **D**: Number of metastases in hot spot foci in liver and lungs from breast cancer bearing mice treated or not treated with P4D. **E**: Area of hot spot foci with metastases in liver and in lungs from 4T1 mice treated or not treated with P4D

The quantitative results of in vivo experiments on 4T1 breast cancer mouse model are presented on Fig. 8A, B, D, and E as graphs with the arithmetic mean values shown as X-cross with the error bars presenting the SD. Due to small sample sizes (n = 8) and the resulting low statistical power of the estimated interferences, the resampling bootstrap technique with 10^6^ iterations and with the assumption, that the tested groups are three times greater (predicted group size n = 24) was used to determine the likelihood of obtaining the revealed differences due to pure chance, followed with Kruskal–Wallis test. Figure 8A shows the number of metastases in liver and lungs observed in the tissue sections from 4T1 mice. Shapiro–Wilk normality W test revealed that the data do not fall upon Gaussian distribution neither for the number of metastases in liver (P-values for Control: 0.0001, for P4D 0.4 mg: < 0.0001, for P4D 4.0 mg: < 0.0001), nor for the ones in lungs (P-values for Control: 0.0037, for P4D 0.4 mg: = 0.0001, for P4D 4.0 mg: < 0.0001). No statistically significant differences between the experimental groups were found for number of metastases in liver (P = 0.7183 by Kruskal–Wallis test) and in lungs (P = 0.0746 by Kruskal–Wallis test). After the implementation of the resampling bootstrap technique the differences between the experimental groups were found to be statistically significant for the number of metastases in lungs (P = 0.0002 by Kruskal–Wallis test), but not in liver (P = 0.7470 by Kruskal–Wallis test). The *post-hoc* Conover-Iman test revealed the significant differences for P4D 0.4 mg vs Control: P = 0.0014; and for P4D 4.0 mg vs Control: P = 0.0006 – the decreased number of metastases in lungs was observed for both P4D-treated groups. The differences between the groups ‘P4D 4.0 mg’ and ‘P4D 0.4 mg’ were not significant (P = 0.7866).

Figure 8B presents the mean values of largest metastasis area in liver and in lungs. As evidenced by Shapiro–Wilk normality W test, the data do not fall upon Gaussian distribution neither for liver (P-values for Control: < 0.0001, for P4D 0.4 mg: = 0.0117, for P4D 4.0 mg: < 0.0001) nor for lungs (P-values for Control: < 0.0001, for P4D 0.4 mg: < 0.0001, for P4D 4.0 mg: 0.0002). Differences between the experimental groups were not statistically significant at n = 8 (P = 0.5095 for liver and P = 0.0867 for lungs by Kruskal–Wallis test). The resampling bootstrap technique application (n = 24; 10^6^ iterations) resulted in the statistically significant differences between the tested groups: P = 0.0402 for area of largest metastasis in liver and P = 0.0351 for area of largest metastasis in lungs by Kruskal–Wallis test.

The number of mice with metastases observed macroscopically did not differ significantly within the tested groups neither for liver, nor for lungs (Fig. 8C, first and second plot from the left). On the other hand, the metastases were observed microscopically in the livers of all mice in each experimental group, whereas the number of mice with metastases observed microscopically in lungs was significantly lower in both P4D-treated groups as compared with the control group (Fig. 8C, first and second plot from the right). The statistical significance was tested by χ^2^ test. The differences between the groups for metastases observed macroscopically were not significant neither for liver (P = 0.3017 for P4D 0.4 mg vs Control; P = 0.3017 for P4D 4.0 mg vs Control; P = 1.000 for P4D 0.4 mg vs P4D 4.0 mg), nor for lungs (P = 1.0000 for P4D 0.4 mg vs Control; P = 0.3017 for P4D 4.0 mg vs Control; P = 0.3017 for P4D 0.4 mg vs P4D 4.0 mg). The differences between the groups for metastases observed microscopically were not significant for liver (P = 1.0000 for P4D 0.4 mg vs Control; for P4D 4.0 mg vs Control; and for P4D 0.4 mg vs P4D 4.0 mg), but were significant for lungs (P = 0.0117 for P4D 0.4 mg vs Control; P = 0.0117 for P4D 4.0 mg vs Control; P = 1.000 for P4D 0.4 mg vs P4D 4.0 mg).

For our study the hot-spot foci were defined as the areas with markedly increased number of metastases counted per 1 mm^2^. The analysis of the number of metastases in hot-spot foci in liver and lungs is shown in Fig. 8D. For the number of metastases in hot-spot foci in liver the data fall upon Gaussian distribution (Shapiro–Wilk normality W test; P values for Control: 0.3236; for P4D 0.4 mg: 0.6140; for P4D 4.0 mg: 0.1838) and the differences between the mean values are not statistically significant (P = 0.3122 by one-way ANOVA for n = 8). The data related with the number of metastases in lungs do not fall upon normal distribution (Shapiro–Wilk normality W test; P values for Control: 0.0012; for P4D 0.4 mg: 0.0002; for P4D 4.0 mg: 0.0002) and the differences between the tested groups are statistically significant, but the differences are on the verge of significance (P = 0.0488 by Kruskal–Wallis test; n = 8). Tukey’s post-test failed to show that the differences are statistically significant: P = 0.1001 for P4D 0.4 mg vs Control; P = 0.1001 for P4D 4.0 mg vs Control; P > 0.9999 for P4D 4.0 mg vs P4D 0.4. The statistical significance was obtained with bootstrap resampling technique (n = 24; 10^6^ iterations; group effect: P < 0.0001 by Kruskal–Wallis test; all pairwise comparisons by Conover-Iman test: P = 0.0004 for P4D 0.4 mg vs Control; P = 0.0004 for P4D 4.0 mg vs Control; P > 0.9999 for P4D 4.0 mg vs P4D 0.4). Thus, upon the application of the resampling bootstrap technique the differences between the experimental groups were found to be statistically significant confirming, that P4D evidently decreased the number of metastases in hot spot foci in lungs (Fig. 8D, right plot).

Figure 8E presents the estimation of the mean area of hot-spot foci with metastases in liver and in lungs. The data do not fall upon Gaussian distribution neither for liver (Shapiro–Wilk normality test; P values for Control: 0.0005; for P4D 0.4 mg: < 0.0001; for P4D 4.0 mg: < 0.0001), nor for lungs (Shapiro–Wilk normality test; P values for Control: 0.0001; for P4D 0.4 mg: < 0.0001; for P4D 4.0 mg: 0.0002). The evident effect of P4D reducing the area of metastases can be seen both in the liver and in the lungs.

However, due to small sample sizes we did not obtain the statistical significance of the observed differences between the experimental groups. The lack of statistical significance between the tested groups was determined by Kruskal–Wallis test at n = 8 (P = 0.2348 for liver; P = 0.0917 for lungs). Therefore, the resampling bootstrap technique was used. Consequently, the differences between the experimental groups were found to be statistically significant with P = 0.0014 for liver and with P = 0.0333 for lungs. The area of hot spot foci was significantly decreased in livers obtained from both P4D-treated groups, while the area of hot spot foci was significantly decreased in lungs only from the mice treated with 4.0 mg injections of P4D.

## Discussion

Triple negative breast cancer (TNBC) is reported to be the most aggressive out of the molecular BC subtypes, with highest mortality rate and worst prognosis [49]. Due to the lack of ER, PR, and eRBB2/HER2 expression, which are the traditional cell surface markers for targeted molecular therapies, the development of a specific therapy for TNBC treatment is challenging [7]. The targeted cancer therapies, often in combination with chemotherapy, have been a subject of a few clinical studies, including poly (ADP-ribose) polymerase (PARP) inhibition, immune checkpoint inhibition, signaling kinases (serine/threonine- or tyrosine-type) inhibition, angiogenesis, epigenetic modifications, and cell cycle [50]. However, these approaches suffer from some limitations, including: high incidence of adverse effects (alveolitis, septic shock, sepsis, hematologic toxicity), acquired drug resistance, lack of suitability for all TNBC patients, and the necessity to be combined with chemotherapy or another medication [50]. Due to the aggressive nature and lack of defined molecular targets, the poor overall survival (OS) of metastatic TNBC has remained essentially unchanged over the past two or three decades, with the median OS of 13 months [50]. Therefore, the development of a new targeted therapy is an urgent task in TNBC management.

A recent report describes that the antibiotic against Gram-positive bacteria Tetrocarcin-A, that was noted for the induction of apoptosis in cancer cells [51], can be a potential novel drug for TNBC treatment due to its antagonistic properties towards F11R/JAM-A [13]. In this study Tetrocarcin-A reduced the growth of TNBC cell lines, both human HCC38 and murine 4T1, and decreased the long-term survival (colony forming ability) of 4T1 cells, that resulted from the decrease of F11R/JAM-A protein level and the subsequent induction of apoptosis via a pathway involving c-FOS-mediated regulation of inhibitor of apoptosis proteins (IAPs). Tetrocarcin-A was also shown to disturb the growth of HER2-positive breast cancer cells by the decrease of F11R/JAM-A protein level [52]. Moreover, the reduction of growth of patient-derived primary breast cancer cells and lung cancer stem cells alongside the F11R/JAM-A protein downregulation was due to cell treatment by Tetrocarcin-A [52]. Likewise, the growth of patient-derived primary TNBC cells and the gross size of 4T1 TNBC cell xenografts in *in ovo/semi *in vivo chicken egg chorionic allantoic membrane (CAM) tumour model was reduced by this antibiotic [13]. Tetrocarcin-A (PubChem CID: 54681516, synonyms: DC-11, Antlermicin A, Antibiotic DC 11, SCHEMBL5478825) that was isolated from *Micromonospora chalcea* subsp. *Kazunoensis* [53], belongs to a class II marine spirotetronate polyketides family of microbial metabolites, commonly produced by marine and terrestrial actinomyces, with potent antitumour and antibiotic properties [54]. This group of spirotetronates contains the spirotetronate motif (tetronic acid spiro-linked to a cyclohexene or cyclohexane ring) integrated within a macrocycle and additionally integrated decalin with the attached oligosaccharide chain [55]. The specificity of the mechanism of F11R/JAM-A downregulation by Tetrocarcin-A appears to be low: it is indirect, acting most probably by inducing lysosomal degradation of F11R/JAM-A [52]. This suggestion was supported by the fact, that the reduction of F11R/JAM-A protein level by Tetrocarcin-A inhibited the growth of all mammary gland epithelial cell lines expressing the high amounts of F11R/JAM-A: not only the cancer cells, but also the non-cancerous MCF-10A line [52] that also expresses high levels of F11R-JAM-A [16]. The early work by Tamaoki et al. describes the antimicrobial mechanism of Tetrocarcin-A based on the inhibition of RNA, cell wall, and protein synthesis, that increases the cell wall permeability in *Bacillus subtilis* and *Escherichia coli* Gram-positive bacteria [56]. However, the detailed mechanism of Tetracarcin-A anticancer activity in eukaryotic cells is not discovered, since its main cellular target is still under investigation [54].

There are several other reports suggesting, that targeting F11R/JAM-A seems to be a promising strategy in TNBC treatment. For example, F11R/JAM-A was reported to be important for self-renewal in TNBC cancer stem cells [21]. Moreover, F11R/JAM-A was implicated in inhibition of TNBC breast cancer cell line MDA-MB-231 invasion by an antibody targeting the tetraspanin CD81 [30]. The limited treatment options for TNBC and the recent reports on F11R/JAM-A role in TNBC prompted us to verify whether F11R/JAM-A cell adhesion molecule can serve as the novel cell surface marker for targeted molecular therapy in TNBC. Therefore, we have performed tests in vitro on cell lines and in vivo on 4T1 breast cancer murine model, whether the F11R/JAM-A peptide antagonist P4D can be efficient when used for the targeted molecular therapy in TNBC. The mechanism of P4D action was thoroughly described previously and was shown to be highly specific by solid phase binding assay, surface plasmon resonance and molecular docking studies [28]. Briefly, peptide 4D mimics the fragment of F11R/JAM-A polypeptide chain responsible for the *trans*-homodimerization of two F11R/JAM-A molecules present on neighboring endothelial cells, thus blocking the tight junction formation de novo, without disrupting the preexisting ones [29]. Moreover, P4D blocks the interactions of F11R/JAM-A molecules relocated upon inflammation from endothelial TJs to the apical endothelial surface with the F11R/JAM-A molecules present on cancer cells, therefore P4D inhibits the early stages of metastasis [24, 29].

Based on online databases analysis, the aberrant expression of F11R/JAM-A cell adhesion molecule is characteristic for most types of cancer (Fig. 1A). This observation is supported by previously published data [57]. The increased level of F11R/JAM-A transcription is notified in breast cancer (Fig. 1B) and is especially apparent in TNBC (Fig. 1C), being a hallmark of poor prognosis due to decreased survival rate (Fig. 1D).

We have previously studied the effect of P4D on the mutual interactions between breast cancer and endothelial cells showing, that P4D inhibited the early stages of metastasis: breast cancer cell adhesion to endothelial monolayer and transendothelial migration of breast cancer cells [29]. In this paper we present the effect of P4D separately on TNBC cells and on endothelial cells. Our data show, that P4D disturbs the cellular interactions within the endothelial monolayer, thus increasing its permeability. This observation is based on the endothelial cell morphology alteration upon treatment with P4D (Fig. 2) and on the decreased TEER of endothelial monolayer (Fig. 6). However, the adhesion and TEM of breast cancer cells was inhibited by P4D as we have previously demonstrated [29]. This is because P4D does not disturb the pre-existing tight junctions, but inhibits only the formation of new tight junctions: this peptide abrogates the TJs formation without the breakdown of endothelial monolayer. Thus the TEER measurements have shown that P4D blocks the endothelial TJs interactions, probably by binding F11R/JAM-A molecules present on endothelial membrane. This in turn inhibits the homophilic interactions between the F11R/JAM-A molecules present on two different neighboring cells. The homophilic F11R/JAM-A interactions are also necessary for TEM of breast cancer cells [58]. The inhibition of breast cancer cell TEM was demonstrated in our previous paper [29], thus the present report is the logical continuation of our previous study.

The antagonistic peptide P4D inhibited the colony forming ability (Fig. 3), viability (Fig. 4), and proliferation (Fig. 5) of TNBC breast cancer cell line MDA-MB-231. This observation supports the previous studies, in which the growth and survival of several cancer cell types, including TNBC cells, was reduced by F11R/JAM-A silencing or pharmacological inhibition of F11R/JAM-A [13, 17, 20]. Of note, the viability and proliferation of endothelial cells were not affected (Figs. 3, 4, 5).

Experiments in vivo were performed using the murine 4T1 metastatic triple negative breast cancer model, which is characterized by spontaneous metastases to distant organs and the development of endothelial dysfunction, that allows the study of a wide aspect of metastasis, including the transmission of cancer cells through the endothelium [59]. We have observed the evident reduction of metastases in both P4D-treated groups of mice, as evidenced by histopathological analysis (Fig. 7A–D). Subsequently, the quantitative analysis of murine samples did not reveal any statistically significant differences between the testes groups (Fig. 8A, B, D, and E). The statistical significance was found only for the number of metastases observed microscopically in lungs, which was evidently lower for both P4D-treated groups (Fig. 8C). The small group size (n = 8) resulted in small statistical power, thus the obtained differences between the groups were not significant. The statistically significant effect of P4D peptide on metastasis inhibition in tested animals was obtained after the application of the resampling bootstrap technique, that was successfully used for data analysis in a previously published study [60] and its use for the data analysis in biomedical field is increasing [61, 62].

Moreover, we have performed the post hoc statistical power analysis by Kruskal–Wallis test to estimate the minimum sample size that guarantees an adequate statistical power. The minimum sample size to offer a sufficient statistical power was determined with Study Size software as to be no smaller than 54–55 mice in each experimental group. There is due to a worldwide scientific issue with an ethical background concerning the reduction of the use of animals for laboratory experiments [63]. Thus, a scientist preparing a request to use the animals for research study is aware, that the smaller is the predicted number of animals for the experiments, the greater is the chance to obtain the permission from an ethics committee for animal research. On the other hand, the small sample size usually results in small statistical power of the estimated interferences. Therefore, the modern statistical approaches, including resampling bootstrap technique, are suitable solutions to avoid the use of a large number of animals for the study. Nevertheless, our findings form the basis for additional studies using larger groups of animals and/or form the basis for additional studies in large animals [64–66].

## Conclusions

Our study is the first scientific report describing the treatment of TNBC with a peptide antagonist of a tight junction molecule and reveals the novel target to treat TNBC, thus opening up the new possibilities to treat the patients suffering from this subtype of breast cancer. The F11R/JAM-A antagonist peptide 4D may be considered as a new potential therapeutic candidate for the treatment of patients suffering from TNBC. The current data extend the results of our previous study [29] showing, that the P4D peptide can efficiently inhibit the early stages of metastasis in vitro, that was demonstrated particularly by the hindered growth and proliferation of TNBC cell line MDA-MB-231. Moreover, in our in vivo experiments the F11R/JAM-A antagonist peptide 4D evidently inhibits the metastasis in the 4T1 breast cancer mouse model. Therefore, our findings can trigger the clinical trials that focus on TNBC treatment with P4D. Alternatively, P4D can also be regarded as a starting point towards the development of a peptidomimetic or a chemically programmed antibody.

### Supplementary Information

**Additional file 1: Figure S1.** Scratch assay results. Scratch area was analysed by ImageJ software with MiToBo ScratchAssayAnalyzer plugin and is expressed as fold change relative to the scratch area at the beginning of the assay (time point: 0 h). The mean values ± SD are shown. The experiment was repeated 4 times (n = 4). **Figure S2.** Spearman correlation between the mass of spleen and the mass of primary tumours dissected from mice used for 4T1 breast cancer model and subjected for treatment with F11R/JAM-A antagonistic peptide 4D. **Table S1.** Summary presenting the detailed data regarding the tumor from each individuals. **Table S2.** Morphological metastases: data from each individual. **Table S3.** Morphological metastases: summary.

**Additional file 2: **.

## Data Availability

The datasets used and analyzed in the current study are available from the corresponding authors on reasonable request.

## Abbreviations

ADC: Antibody drug conjugate
ANOVA: Analysis of variance
ATCC: American type culture collection
BC: Breast cancer
BRCA: Breast invasive carcinoma
BrdU: 5-Bromo-2ʹ -deoxyuridine
CAM: Chorionic allantoic membrane
CFA: Colony forming assay
CSC(s): Cancer stem cell(s)
CTRL; Ctrl: Control (non-treated) sample of a cell line
DMEM: Dulbecco’s modified Eagle’s medium
EDTA: Ethylenediaminetetraacetic acid
EGA: European genome-phenome archive
EGF: Epidermal growth factor
ELISA: Enzyme-linked immunosorbent assay
ER: Estrogen receptor
F11R: F11-receptor
FBS: Fetal bovine serum
FFPE: Formalin-fixed, paraffin-embedded
GEO: Gene Expression Omnibus
GMM: Genetically modified microorganisms
HAT: 100 M hypoxanthine, 0.4 M aminopterin, and 16 M thymidine
HER2/erbB2: Human epidermal growth factor receptor 2
HMEC-1: Human microvascular endothelial cells 1
IAP(s): Inhibitor of apoptosis proteins
JAM-A: Junctional adhesion molecule-A
KM plotter: Kaplan–Meier plotter
MAb: Monoclonal antibody
miR-145: MicroRNA 145
MTT: 3-(4,5-Dimethylthiazol-2-yl)-2,5-diphenyltetrazolium bromide
Num.: Number
OS: Overall survival
P4D: F11R/JAM-A antagonistic peptide 4D
PARP: Poly (ADP-ribose) polymerase
PBS: Phosphate buffered saline
PD-L1: Programmed cell death ligand 1
PE: Plating efficiency
PI3K: Phosphoinositide-3-kinase
PKB/Akt: Protein kinase B/Akt
PR: Progesterone receptor
RNA-Seq: RNA sequencing
Scr: Control peptide with the scrambled sequence of P4D
SD: Standard deviation
SF: Surviving factor
TCGA: The cancer genome atlas
TEER: Transepithelial/endothelial electrical resistance
TEM: Transendothelial migration
TJ(s): Tight junctions(s)
TNBC: Triple-negative breast cancer
TNMPlot: Differential gene expression analysis in tumor, normal and metastatic tissues
Trop-2: Trophoblast cell-surface antigen-2
UALCAN: The University of Alabama at Birmingham Cancer data analysis Portal
