# Supplementary material for: Triple negative breast cancer metastasis is hindered by a peptide antagonist of F11R/JAM‑A protein
Source: Cancer Cell Int. 2023 Aug 11;23:160. doi: 10.1186/s12935-023-03023-4 (PMC10416405; doi:10.1186/s12935-023-03023-4)
Supplement: Supplementary file 1 — Additional file 1: Figure S1. Scratch assay results. Scratch area was analysed by ImageJ software with MiToBo ScratchAssayAnalyzer plugin and is expressed as fold change relative to the scratch area at the beginning of the assay (time point: 0 h). The mean values ± SD are shown. The experiment was repeated 4 times (n = 4). Figure S2. Spearman correlation between the mass of spleen and the mass of primary tumours dissected from mice used for 4T1 breast cancer model and subjected for treatment with F11R/JAM-A antagonistic peptide 4D. Table S1. Summary presenting the detailed data regarding the tumor from each individuals. Table S2. Morphological metastases: data from each individual. Table S3. Morphological metastases: summary. [file 12935_2023_3023_MOESM1_ESM.doc]

**Additional file material**

**Triple Negative Breast Cancer metastasis is prevented by a peptide antagonist of F11R/JAM‑A protein**

Radosław Bednarek^1^, Dagmara W. Wojkowska^2^, Marcin Braun^3^, Cezary Watala^2^, Moro O. Salifu^4^, Maria Swiatkowska^1*^, Anna Babinska^4*^

^1^Department of Cytobiology and Proteomics, Medical University of Lodz, Lodz, Poland

^2^Department of Haemostasis and Haemostatic Disorders, Medical University of Lodz, Lodz, Poland

^3^Department of Pathology, Chair of Oncology, Medical University of Lodz, Lodz, Poland

^4^Department of Medicine, State University of New York, Downstate Medical Center, Brooklyn, NY, USA

^*^these Authors contributed equally in this work

Corresponding author:

Radoslaw Bednarek, PhD, Department of Cytobiology and Proteomics, Chair of Biomedical Sciences, Medical University of Lodz, ul. Mazowiecka 6/8, 92-215 Lodz, Poland, email: radoslaw.bednarek@umed.lodz.pl

Tel.: +48 42 2725720, Fax: +48 42 2725730

**Additional file material and Methods**

**Scratch assay**

The EA.hy926, HMEC-1, and MDA-MB-231 cells were untreated (Control), treated with the control scrambled peptide (Scr), or with the F11R/JAM-A antagonistic peptide (P4D) at a concentration of 500 μM at the time of cell seeding in 6-well plates. Scratches were made with a 200-μl pipette tip. The cells were incubated in a humidified incubator under 5% (v/v) CO_2_ conditions at 37 °C. Microphotographs were taken at the following time points: 0, 3, 6, 12, 24 hours (Olympus CKX41 at phase contrast with 400× magnification, equipped with Olympus C-5050 digital camera and Olympus C3040-ADU adapter port tube). Scratch area analysis was performed by ImageJ software with MiToBo ScratchAssayAnalyzer plugin. Statistical analysis and data plotting was performed with GraphPad Prism 6.01. As estimated by Shapiro-Wilk normality test the data do not fall upon Gaussian distribution. Friedman test followed by Dunn’s multiple comparisons did not detect statistically significant differences between the scratch areas on the microphotographs taken at the time specified points above (n = 4). The plots below represent the fold change (arithmetic means ± SD) of the scratch areas measured by the software in pixels with relation to the areas at the “0 hrs” time point (= 1.0).

**Additional file Figures**

Figure S1. Scratch assay results. Scratch area was analysed by ImageJ software with MiToBo ScratchAssayAnalyzer plugin and is expressed as fold change relative to the scratch area at the beginning of the assay (time point: 0 hours). The mean values ± SD are shown. The experiment was repeated 4 times (n = 4).

Figure S2. Spearman correlation between the mass of spleen and the mass of primary tumours dissected from mice used for 4T1 breast cancer model and subjected for treatment with F11R/JAM-A antagonistic peptide 4D.

**Additional file Tables**

Table ST1. Summary presenting the detailed data regarding the tumor from each individuals.

| *No.* | *ID* | *Tumor size [mm X mm]* | *HER2 (0,1: negative; 2,3: positive)* | *Remarks* |
| --- | --- | --- | --- | --- |
| 14 | gr I 1 | 12.5x7.5 | 0 |  |
| 13 | gr I 2 | 11x9 | 0 |  |
| 18 | gr I 3 | 10x5.5 | 0 | significant necrosis |
| 17 | gr I 4 | 11x7.5 | 0 |  |
| 3 | gr I 5 | 12x9 | 0 |  |
| 2 | gr I 6 | 16x12 | 1 |  |
| 1 | gr I 7 | 8x6.5 | 0 |  |
| 6 | gr I 8 | 10.5x8.5 | 0 |  |
| 5 | gr II 1 | 12x10 | 0 | significant necrosis |
| 4 | gr II 2 | 6.5x3 | 0 | small tumor |
| 9 | gr II 3 | 13x7 | 0 |  |
| 8 | gr II 4 | 14x7 | 0 |  |
| 7 | gr II 5 | 11x9 | 0 | significant necrosis |
| 12 | gr II 6 | 14x6.5 | 0 |  |
| 11 | gr II 7 | 10x7.5 | 1 |  |
| 10 | gr II 8 | 11x8 | 0 |  |
| 21 | Ctrl1 | 9x5 | 2 | strong overexpression of F11R/JAM-A |
| 20 | Ctrl2 | 8x5 | 0 |  |
| 19 | Ctrl3 | 14x11 | 2 | strong overexpression of F11R/JAM-A |
| 24 | Ctrl4 | 15x10 | 0 | significant necrosis |
| 23 | Ctrl5 | 12x8 | 0 |  |
| 22 | Ctrl6 | 13x10 | 0 |  |
| 16 | Ctrl7 | 11x9.5 | 0 |  |
| 15 | Ctrl8 | 22X12 | 1 | significant necrosis |

Table ST2. Morphological metastases: data from each individual.

| *ID* | *Organ* | *Metastases macroscopically*  *(1: yes;*  *0: no)* | *Metastases microscopically*  *(1: yes;*  *0: no)* | *Area of the biggest metastasis in a sample [mm^2^]* | *Cross-sectional area of the whole sample [mm^2^]* | *Number of metastases in a sample* | *Number of metastases per 1 mm^2^ of cross-section in hot-spot foci* | *% of hot-spot area covered by metastases* |
| --- | --- | --- | --- | --- | --- | --- | --- | --- |
| P4D 4 1 | liver | 0 | 1 | 0.2700 | 198.00 | >50 | 3 | 29 |
| P4D 4 1 | lung | 0 | 1 | 0.0660 | 62.00 | 5.00 | 2 | 12 |
| P4D 4 2 | liver | 0 | 1 | 0.0035 | 202.00 | >50 | 3 | <5 |
| P4D 4 2 | lung | 0 | 0 | 0.0000 | 63.00 | 0.00 | 0 | 0 |
| P4D 4 3 | liver | 0 | 1 | 0.0070 | 248.00 | >50 | 7 | <5 |
| P4D 4 3 | lung | 0 | 0 | 0.0000 | 65.00 | 0.00 | 0 | 0 |
| P4D 4 4 | liver | 0 | 1 | 0.0060 | 231.00 | >50 | 9 | <5 |
| P4D 4 4 | lung | 0 | 0 | 0.0000 | 65.00 | 0.00 | 0 | 0 |
| P4D 4 5 | liver | 0 | 1 | 0.0050 | 243.00 | >50 | 10 | <5 |
| P4D 4 5 | lung | 0 | 0 | 0.0000 | 58.00 | 0.00 | 0 | 0 |
| P4D 4 6 | liver | 0 | 1 | 0.0065 | 261.00 | >50 | 15 | 5 |
| P4D 4 6 | lung | 0 | 0 | 0.0000 | 61.00 | 0.00 | 0 | 0 |
| P4D 4 7 | lung | 0 | 0 | 0.0000 | 48.00 | 0.00 | 0 | 0 |
| P4D 4 7 | liver | 0 | 1 | 0.0040 | 243.00 | 20.00 | 3 | <5 |
| P4D 4 8 | liver | 0 | 1 | 0.0040 | 239.00 | >50 | 14 | 10 |
| P4D 4 8 | lung | 0 | 1 | 0.0900 | 68.00 | 5.00 | 1 | 9 |
| gr II 1 | liver | 0 | 1 | 0.0040 | 223.00 | >50 | 6 | <5 |
| gr II 1 | lung | 0 | 0 | 0.0000 | 64.00 | 0.00 | 0 | 0 |
| gr II 2 | liver | 0 | 1 | 0.0035 | 216.00 | 25.00 | 2 | <5 |
| gr II 2 | lung | 0 | 0 | 0.0000 | 58.00 | 0.00 | 0 | 0 |
| gr II 3 | liver | 0 | 1 | 0.0090 | 249.00 | >50 | 17 | 10 |
| gr II 3 | lung | 0 | 1 | 0.1350 | 63.00 | 8.00 | 2 | 28 |
| gr II 4 | lung | 0 | 0 | 0.0000 | 49.00 | 0.00 | 0 | 0 |
| gr II 4 | liver | 0 | 1 | 0.0180 | 228.00 | >50 | 11 | <5 |
| gr II 5 | liver | 0 | 1 | 0.0055 | 242.00 | >50 | 15 | 5 |
| gr II 5 | lung | 0 | 0 | 0.0000 | 64.00 | 0.00 | 0 | 0 |
| gr II 6 | liver | 0 | 1 | 0.0060 | 235.00 | >50 | 16 | 5 |
| gr II 6 | lung | 0 | 0 | 0.0000 | 53.00 | 0.00 | 0 | 0 |
| gr II 7 | liver | 0 | 1 | 0.0025 | 230.00 | >50 | 7 | <5 |
| gr II 7 | lung | 0 | 0 | 0.0000 | 64.00 | 0.00 | 0 | 0 |
| gr II 8 | liver | 0 | 1 | 0.0050 | 287.00 | >50 | 12 | <5 |
| gr II 8 | lung | 1 | 1 | 2.1500 | 72.00 | 7.00 | 1 | 100 |
| Ctrl1 | liver | 0 | 1 | 0.0025 | 252.00 | 24.00 | 4 | <5 |
| Ctrl1 | lung | 0 | 0 | 0.0000 | 65.00 | 0.00 | 0 | 0 |
| Ctrl2 | liver | 0 | 1 | 0.0035 | 238.00 | 20.00 | 3 | <5 |
| Ctrl2 | lung | 0 | 1 | 0.0030 | 64.00 | 1.00 | 1 | <5 |
| Ctrl3 | liver | 0 | 1 | 0.0120 | 170.00 | >50 | 8 | 8 |
| Ctrl3 | lung | 0 | 1 | 0.0030 | 54.00 | 1.00 | 1 | <5 |
| Ctrl4 | liver | 0 | 1 | 0.0050 | 241.00 | >50 | 11 | <5 |
| Ctrl4 | lung | 0 | 1 | 0.0012 | 65.00 | 1.00 | 1 | 2 |
| Ctrl5 | liver | 0 | 1 | 0.0065 | 253.00 | >50 | 14 | 5 |
| Ctrl5 | lung | 0 | 1 | 0.0055 | 68.00 | 3.00 | 1 | <5 |
| Ctrl6 | liver | 0 | 1 | 0.0850 | 268.00 | >50 | 12 | 10 |
| Ctrl6 | lung | 0 | 1 | 0.2950 | 56.00 | 3.00 | 1 | 30 |
| Ctrl7 | liver | 1 | 1 | 16.0000 | 201.00 | >50 | 1 | 100 |
| Ctrl7 | lung | 0 | 1 | 0.0090 | 61.00 | 9.00 | 3 | <5 |
| Ctrl8 | lung | 1 | 1 | 6.0100 | 72.00 | 19.00 | 1 | 100 |
| Ctrl8 | liver | 0 | 1 | 0.4500 | 228.00 | >50 | 2 | 55 |

Table ST3. Morphological metastases: summary.

| *Group* | *Number of metasases macroscopically* | *Number of metasases microscopically* | *Mean area of the biggest metastasis in a sample [mm^2^]* | *SD of the area of the biggest metastasis in a sample [mm^2^]* | *Mean cross-sectional area of the whole sample [mm^2^]* | *SD of the cross-sectional area of the whole sample [mm^2^]* | *>50 metastases in a sample [N]* | *Mean number of metastases per 1 mm^2^ of cross-section in hot-spot foci* |
| --- | --- | --- | --- | --- | --- | --- | --- | --- |
| P4D 4 | 0 | 10 | 0.0289 | 0.07 | 147.19 | 90.1341362 | 7 | 4.1875 |
| P4D 0.4 | 1 | 10 | 0.1462 | 0.54 | 149.81 | 93.2174653 | 7 | 5.5625 |
| Control | 2 | 15 | 1.4307 | 4.16 | 147.25 | 89.6485731 | 6 | 4.0000 |
